# *Streptococcus pyogenes* carriage is associated with distinct *in vivo* transcriptional states but not carrier-specific genotypes

**DOI:** 10.1016/j.celrep.2026.117925

**Published:** 2026-08-31

**Authors:** Nicholas Faiola, Jason M. Wood, Nicole Casab, May Xu, Kevin Quirk, Christopher Thomas, Eliza Klos, Jordan Thesier, Mitchell Waldran, Mark Maienschein-Cline, Michael J. Federle, Gregory P. DeMuri, Ellen R. Wald, Laura C. Cook

**Affiliations:** 1 Department of Biological Sciences, Binghamton Biofilm Research Center, Binghamton University, Binghamton, NY, USA; 2 Research Informatics Core, Research Resources Center, University of Illinois at Chicago, Chicago, IL, USA; 3 Department of Medicinal Chemistry and Pharmacognosy, University of Illinois at Chicago, Chicago, IL, USA; 4 Department of Pediatrics, Division of Infectious Diseases, University of Wisconsin School of Medicine and Public Health, Madison, WI, USA; 5 Lead contact

## Abstract

Group A *Streptococcus* (GAS) is a major human pathogen causing extensive global morbidity. While the asymptomatic carrier state challenges clinical management and contributes to community transmission, genomic, and transcriptomic analyses of naturally acquired human GAS carriage have been lacking. In this study, samples are collected from individuals before and after treatment for acute GAS pharyngitis. Selected isolates undergo whole-genome sequencing and RNA sequencing, providing transcriptional profiling of GAS directly from human-derived oropharyngeal swabs. Genomic analysis reveals no consistent carrier-specific genotypes, and *in vitro* assays show no major differences in biofilm formation or antibiotic susceptibility between carrier and non-carrier isolates. However, *in vivo* transcriptional profiling identifies distinct gene expression patterns when comparing acute infection and post-treatment carriage phases, though acute-phase profiles do not predict treatment failure. These findings suggest GAS carriage is influenced by both bacterial traits and host factors, advancing our understanding of persistence and transmission.

## In brief

Using paired genomic and direct *in vivo* transcriptomic sequencing of human oropharyngeal swabs, Faiola et al. demonstrate that GAS carriage is defined by unique phase-specific gene expression patterns. The results highlight a complex, mechanism of bacterial persistence, and transmission rather than conserved carriage genotypes.

## Graphical abstract

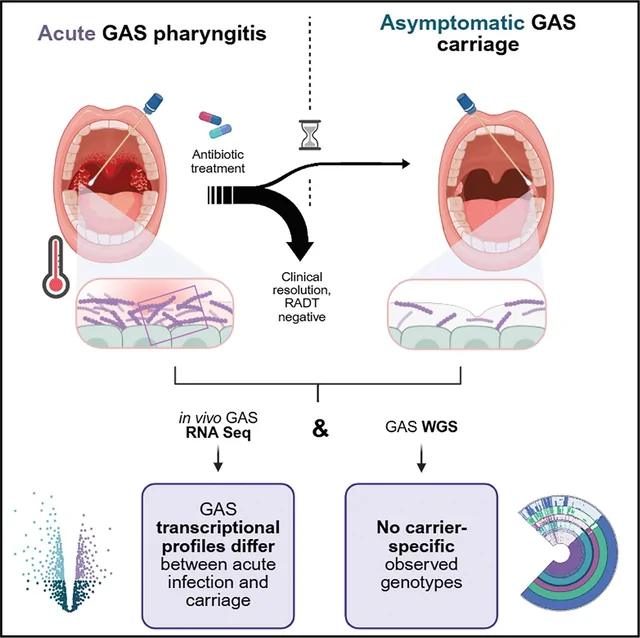

## INTRODUCTION

Many species of bacteria that colonize the human upper respiratory tract maintain dichotomous lifestyles, capable of both mutualistic and pathogenic relationships with the host. Species such as *Staphylococcus aureus*, *Streptococcus pneumoniae*, and *Streptococcus pyogenes* (Group A *Streptococcus*, GAS) cause considerable clinical and economic burdens due to frequent infections; yet these species are carried asymptomatically in the upper respiratory tract of the human population at high rates. GAS is the most common cause of bacterial pharyngitis in children and adults, causing both suppurative (acute otitis media, acute sinusitis, mastoiditis, musculoskeletal, and systemic infections) and non-suppurative (post-streptococcal glomerulonephritis and rheumatic fever) sequelae. Every year, over 600 million new cases of GAS pharyngitis occur globally.^1^ Rates of symptomatic GAS pharyngitis vary between 15%–30% for children and 4%–10% in adults in the developed world, with even higher rates in developing countries.^1–6^ In the last decade, there has been a documented re-emergence of both superficial and invasive *S*. *pyogenes* infections worldwide.^7–9^ Most superficial GAS infections are cleared spontaneously or after appropriate antibiotic administration; however, in many children, GAS is part of the normal pharyngeal microbiota, known as a GAS carrier state. The carrier state of GAS is defined as the presence of the organism in the pharynx without evidence of inflammation or increased serological response to extracellular antigens.^10,11^ Pharyngeal GAS colonization rates in asymptomatic school-aged children vary based on location but are most often estimated to be between 8%–20%.^4,12–15^ Several studies in which children have been followed longitudinally demonstrate that the duration of carriage is variable, but children have been shown to carry the same strain of GAS for months to years.^15,16^

It has been proposed that the true streptococcal carrier state can be most accurately determined retrospectively as antimicrobial treatment failure following an acute infection,^17^ and several studies have used treatment failure to define GAS carriers.^18–20^ In clinical practice, it is proposed that doctors should suspect a child is a carrier if they present with closely spaced recurrences of pharyngitis with microbiologic confirmation of GAS. It has been suggested that a reliable way to confirm this is to perform a throat culture shortly after the child completes an effective round of antibiotics. If the asymptomatic child has no symptoms but the test is still positive, carriage should be suspected.^11^

The carrier state is of particular significance to public health because carriers may contribute to the spread of GAS to others in the population. Carrier isolates could harbor accessory genetic information that could be acquired through horizontal transfer to co-colonizing strains and species. GAS carriage has been associated with community outbreaks of pharyngitis^21^ and fatal invasive disease in close contacts,^22^ and treatment failure has been implicated in development of post-infectious sequelae such as acute rheumatic fever.^23^ In addition, the carrier state is a confounder for clinicians. Patients with viral pharyngitis who are also carriers may test positive for GAS, leading to antibiotic administration and an estimated 1.5 million unnecessary antibiotic courses annually in the United States.^17^ It is also likely that upper airway inflammation associated with symptoms of viral pharyngitis may promote GAS transmission by increasing bacterial shedding from colonized individuals.

Previous studies have found that some cultured GAS isolates recovered from asymptomatic individuals contained genetic polymorphisms that, when integrated into a virulent isolate, caused a carrier-like phenotype in animal models.^24–26^ These findings point to asymptomatic carriage being the result of genetic mutations in the pathogen, potentially arising during virulent episodes, that effectively diminish virulence but maintain survival in the host, at least temporarily. This contradicts conventional thinking in relation to other pathobionts like *S*. *aureus* and *Haemophilus influenzae*, in which the carried bacterium is thought to be the evolutionary ideal due to survival advantage.^27–31^ It is therefore important to thoroughly test these alternative hypotheses using naturally occurring GAS isolates derived from human patients determined to be carriers.

As a human-restricted pathogen, suitable animal models for GAS pharyngitis are limited and their relevance is often uncertain. Macaque^32,33^ and murine^34,35^ studies have been used to characterize transcriptional patterns of GAS strains associated with acute inflammatory disease; however, comparable investigations in humans have not been performed. Recently, a human challenge trial examining interactions between GAS and the human mucosa has been undertaken, representing an important advance in the field.^36,37^ To date, however, no bacterial transcriptional data have been reported from GAS isolated directly from human patients.

Here, we describe a collection of samples from human subjects treated for acute streptococcal pharyngitis obtained both before and after antibiotic treatment. We performed whole genome sequencing (WGS) and transcriptional profiling of selected isolates, comparing patient swabs that were cleared of GAS by treatment (acute, non-carrier patients on the initial visit), with those that remained colonized after treatment (carriers, on visit 1 and on follow-up visit 2). WGS did not identify conserved carrier-specific genotypes although SNPs were observed in carrier isolates. *In vitro* analysis of biofilm formation and antibiotic resistance profiles showed no major differences between non-carrier and carrier isolates. Transcriptional profiling of GAS recovered directly from the oropharynx during infection, relative to liquid culture, reveals distinct metabolic and virulence-associated expression programs characteristic of the host environment. Notably, gene expression differed between GAS isolates recovered during acute infection (visit 1) and those persisting after antibiotic treatment (visit 2), providing a carriage-associated transcriptional signature. In contrast, transcriptomic comparisons of carrier and non-carrier isolates during the acute phase of infection (both at visit 1) did not identify expression patterns predictive of subsequent asymptomatic colonization. Together, these findings provide essential insight into GAS acute infection and carriage potential and suggest a dominant role for host factors and/or host genetic variation in determining GAS persistence following antibiotic therapy. This is consistent with clinical data in the school setting which shows that carriers are likely to become carriers again in subsequent years and frequently switch *emm* types.^15^

## RESULTS

### Collection of longitudinal patient samples

A flow chart of the sample collection process is shown in Figure 1. Samples used in this study were collected from 205 children recruited from four pediatric clinics in Madison, WI between 2012–2023 (Figure 1). Eligibility criteria included age between 5 and 15 years, a diagnosis of acute pharyngitis, and having not been treated with antibiotics in the last 30 days. Children were excluded from this study if they had an allergy to β-lactam antimicrobials, as described further, or if they were treated with an antibiotic other than a β-lactam.

Children presented to the clinics with acute symptoms of sore throat (visit 1, labeled −1). A swab was obtained from the posterior pharynx (tonsillar pillars and posterior pharyngeal wall) of the patient. A rapid antigen detection test (RADT) was used for initial diagnosis. If the RADT was positive, the child and family were informed by the research staff that the child was eligible for the study and if the family and patient agreed to participate, signed informed consent was obtained, and two additional throat swabs were collected. One swab was stored immediately at −80°C while another was forwarded to the laboratory to culture for GAS using standard methods and the cultured isolate preserved. Treatment of the patient was undertaken with an appropriate dose of either amoxicillin or penicillin V for up to 10 days at the discretion of the provider. Additional swabs were obtained approximately 26–28 days after the first visit (visit 2, labeled −2). If the visit 2 follow-up swab was negative for GAS on culture, the patient was deemed to have presented with an acute infection cleared by antibiotics (labeled as non-carrier, NC throughout). These follow up swabs were not transcriptionally or genomically analyzed in this study as they were GAS negative. If visit 2 swab was still positive for GAS by culture and the children were asymptomatic without signs of pharyngeal inflammation, the subject was deemed as a carrier (labeled as carrier, C or Car throughout) and the remaining swab was again stored at −80°C. All longitudinal cultured isolates from carriers on visits 1 and 2 were tested by PCR and Sanger sequencing to demonstrate concurrent *emm* types between the first and second visit swabs to preclude new infections by different GAS serotypes. A single isolate was banked from each swab. The strains isolated and further examined here are described in Table 1. Each isolate was marked as non-carrier or carrier, and were additionally marked as being collected on the initial visit (swab labeled −1) or the return visit ~4 weeks later (swab labeled −2). The clinical characteristics of enrolled patients are shown in Table S1. Clinical data were available for 11 of the 13 carriers and 163 of the 168 non-carriers. The signs and symptoms in carriers vs. acute non-carriers were similar with the exception of headache and cough that were more common in carrier subjects than non-carriers. Serology was not completed on patients enrolled in this study.

### Antibiotic resistance profiles

The minimum inhibitory concentration of four antibiotics was examined for 40 non-carrier and 13 carrier strains using Liofilchem antibiotic test strips on blood agar plates (Table S2). As expected, all strains were identified as susceptible to penicillin and amoxicillin, the antibiotics used clinically for treatment of patients in this study, indicating that antibiotic resistance did not arise in the carrier strains even in those that remained following a course of antibiotics. Three of the collected isolates, C2, C10, and NC46, showed resistance to erythromycin. One strain, NC39, was resistant to clindamycin. No resistance to antibiotics developed following β-lactam usage in the carrier isolates and we did not observe mutations arising in any penicillin binding protein genes.

### *emm* type distribution

80 cultured strains, 67 non-carrier, and 13 carrier isolates were assessed for *emm* type using PCR and Sanger sequencing as described by the CDC.^38^ All carrier visit 2 isolates (*n* = 4) had identical *emm* sequences to the initial visit 1 isolates so only one sample was included in *emm* type analysis to not skew the results. The distribution of *emm* types in these isolates are shown in Figure S1A. In this small cohort of samples, non-carrier isolates had a wider range of *emm* types and a more even spread than carrier isolates. The percent of carrier isolates with *emm* type 6 was significantly higher than expected compared to the non-carrier isolate data and the majority of the carrier isolates (9 of 13) were either *emm* type 6 or 89. Over the years of sample collection, we observed the *emm* type distribution changed somewhat but not significantly for any particular *emm* type or year block (Figure S1B), suggesting that most of these *emm* types were circulating in the population over the entire course of the experiment. Due to the low sample size collected during the COVID-19 pandemic, we combined samples collected between 2018 and 2023.

### In vitro biofilm formation is not correlated with carriage propensity

Bacterial biofilm formation is associated with numerous chronic infections and carriage of organisms on the mucosa. It has been proposed that propensity to form biofilms is associated with GAS carriage in the tonsils^39^ and that treatment failure may be a result of increased biofilm formation.^40^ To determine whether increased *in vitro* biofilm formation was seen in the carrier or non-carrier isolates, we tested the ability of 44 non-carrier and 13 carrier isolates to form biofilms *in vitro* using a microtiter dish assay with a reference strain (NZ131) for comparison in each assay. Biofilm formation varied widely between isolates from very little biofilm formation to high biofilm formation, but there was no significant trend regarding differences in biofilm formation between carrier and non-carrier isolates (Figure S2B). We also did not observe any trends for particular *emm* types to have higher or lower biofilm formation (Figure S2A).

### Genomic conservation in carrier and non-carrier strains

Selected isolates (*n* = 31; 18 non-carrier samples, 11 carrier strains with an additional 2 sequenced visit 2 isolates) were submitted for both short (Illumina) and long (PacBio) read WGS. Isolates were selected for WGS in certain *emm* groups to allow for easier comparison across isolates of different *emm* serotypes. Figure 1 is a circular plot showing comparative genomics for sequenced isolates including *emm* type concordant reference genomes. The isolate pan-genome contains a total of 2,284 genes of which ~60% (1,395) made up the single-copy “core genome”. The remaining ~40% (889 genes) make up the accessory genome containing inter- and intra-*emm* type variation among the isolates (Figure 1). The accessory genome of these isolates is made up of a large percentage of mobile genetic elements including many phage-related genes and transposons. Many of the genes that fall into the unknown function category also appear to be phage-related based on genome location (Figure S3A). The Anvi’o pangenome visualization provides a spatially organized comparison of gene distribution across all strains. By clustering genomes based on their gene frequency signatures, we can visually observe that the presence or absence of accessory genes aligns largely with serotype designations rather than phenotypic categories of carrier or non-carrier. Genomic architecture and gene distribution align tightly by *emm* serotype rather than the clinical phenotype of carrier versus non-carrier, making serotype the strongest predictor of genomic structure.

A notable exception to this pattern is isolate NC-37. Interestingly, while NC-37 has the *emm1* gene allele, and its accessory genome lines well with the rest of *emm1* isolates (Figure 1), many of the genes in the core genome align better with *emm89* serotypes strains. The *emm1*-like core genes are interspersed throughout the genome rather than contained in a single recombination island. Mixed population analysis ruled out the hypothesis that chimeric assemblies derived from mixed cultures explained the anomalous positions of NC-37. Variant calling across all reference mappings revealed zero positions within the intermediate 5%–95% allele frequency range, confirming single-genome purity. Furthermore, IQ-TREE 2 phylogenetic reconstruction was highly concordant with the FastTree topology and NC-37 clades just outside the *emm89* serogroup. High bootstrap support (99%–100%) at key nodes confirmed this placement was not an artifact of the chosen tree-search algorithm or substitution model. Collectively, these data demonstrate that the anomalous placement represents a genuine biological signal.

No major genomic variations clearly differentiated carrier from non-carrier isolates. We examined amino acid sequences for some known GAS virulence and carriage factors^24–26,41^ in our sequenced samples to determine whether particular allele presence was associated with carrier or non-carrier isolates (Figure 2). Genetic polymorphisms identified in the carrier strains remained identical between the patients’ first and second visits (−1 and −2 isolates). The exception was a confirmed HasB truncation that appeared exclusively in the visit 2 isolate of patient Car13 (described further). Because of this consistency across all other matched samples, the data for visits 1 and 2 were consolidated into a single line per patient in Figure 2 rather than being listed separately. Toxin allele variants of SmeZ, SpeA, SpeI, SpeC, SpeJ, SpeG, SpeH, and Ssa were determined based on previous literature.^42^ All strains contained between 3–5 toxin genes and no SNPs or indels were observed in these genes differentiating isolates from others of that same *emm* type. Truncations in SpeH were found in four of the five *emm6* isolates but these were not limited to carrier isolates. Our data did not reveal an obvious correlation between toxin gene presence and carrier or non-carrier isolates but with *emm* type, as expected. The presence or absence of the remaining selected virulence factors was almost completely *emm*-type dependent and conservation of protein sequences matched in a serotype-specific manner rather than aligning by carriage versus acute designations.

SNPs or truncations in GAS genes such as *liaS*,^25^ *hasA*,^41^ and *sclA*^26^ have been previously associated with carriage propensity using animal models of GAS colonization. None of the specific previously identified mutations were found in any of our carrier or non-carrier isolates; although one carrier (C13) did have a nucleotide insertion causing a frameshift in SclA (Table 2), one carrier and one non-carrier isolate had mutations or truncations within HasA, and one carrier isolate had a truncation in HasB2 (Figure 2). Similarly, while a *mga* promoter deletion has previously been shown to induce a carrier-like phenotype,^24^ the promoter deletions found in this study were strictly *emm*-type dependent and present in both carrier and non-carrier strains (Figure S4). A few novel amino acid polymorphisms were present only in carrier isolates from our study (Table 2). Interestingly, one such polymorphism was found in two separate carrier isolates, C4 and C10 (both *emm*6) resulting in an S364F change in DltD. This warrants further investigation to determine if this change results in differential activity of DltD and if affects carriage propensity.

### Comparative genomics of carrier patient sample over time

Besides comparing all isolates as a group, we also compared genome sequences of two of the longitudinal carriage isolates before and after antibiotic treatment. C3–1 and C3–2 differed by two SNPs resulting in a Phe139Leu change in the gene *aapA (spy_1654)* and a T to G nucleotide at position 1,428,166 change in a non-protein coding region between the stop codons of adjacent genes. Additional differences such as duplications were seen in the initial assembly but, when the genomic DNA was checked with Sanger sequencing in regions that did not match, the mismatches were shown to be the result of sequencing or assembly error. Corrected sequences for C3–1 and C3–2 are available as GenBank files through NCBI’s GenBank database (Table 1).

C13–1 and C13–2 had two SNPs in rRNA genes; G to A at position 1741 and T to C at position 2969. C13 isolates contain a gene found in some sequenced isolates of GAS and present in our *emm6* isolates, annotated as a putative LPXTG adhesin homologous to M6_spy1173 (*smc*). In MGAS10394, this 1123 amino acid protein contains three full C-terminal repeats of 91 amino acids. In C13–1, this protein contains eight of these repeats and C13–2 contains only nine repeats (Figure S5), resulting in the difference in genome size (Table 1). The function of this protein is currently unknown. Additionally, isolate C13–2 contained a nucleotide insertion of an additional A at position 1,271,084, resulting in a truncated HasB. C13–2 retains intact HasB.2 and HasC.2, which can independent support capsule production in the absence of functional HasB.^43^ Because capsule levels can affect intracellular invasion and carriage, we quantified capsule production across isolates with capsule gene mutations. No significant differences in capsule levels were observed between isolates C13–1 (*emm6*, 23.9 ± 4.5 fg/CFU, *n* = 3) and C13–2 (*emm6*, 22.7 ± 9.0 fg/CFU). Similarly, isolates C6 (*emm1*, 13.0 ± 6.5 fg/CFU) and NC8 (*emm12*, 13.6 ± 6.4 fg/CFU) both had detectable capsule levels similar to other isolates of the same *emm* type (Figure S6A). Consistent with these capsule phenotypes, intracellular invasion assays revealed only a minor, non-significant increase in C13–2 invasion compared to C13–1 (Figure S6B). More broadly, both carrier and non-carrier isolates displayed a wide range of invasion efficiencies, with carrier strains distributed across both the high and low ends of the invasion spectrum.

### Transcriptional patterns of GAS strains during carriage and acute infection differentiate between disease states but are not predictive of future treatment failure and carriage

To perform transcriptomics on GAS isolates of different serotypes, we first created a FASTA database file containing all coding sequences (CDS) from eight GAS reference genomes (referred to as our transcriptomic pan genome). 6 *emm* types (1, 2, 4, 6, 12, 28, and 89) were represented, making up >87% of the *emm* type diversity found in our cohort. The final open reading frame (ORF) count in our transcriptomic pan-genome was 2,365 (1,196 core and 1,169 accessory genes), making it approximately 35% larger than the average single GAS genome (1,717 genes^44^). Table S3 shows the transcriptomic pan-genome along with amino acid sequence of translated open reading frames. The genes are indicated to be part of the core (uncolored) or accessory (shaded blue) genome in all supplemental tables. Like the pan-genome created from our isolate sequencing data, the accessory gene content in the transcriptomic pan-genome contains a high percentage of genes with unknown function and genes known to be part of the mobilome (Figure S3B).

We performed RNA-sequencing on swabs collected directly from patients during an acute infection. Comparisons were made between different groups as described further. In total, transcriptional profiling was done on 28 non-carrier swabs (all visit 1), 9 carrier swabs (5 during the acute phase visit 1 and 4 from the return visit 2), and 21 liquid cultures as comparators. Not all of the swab samples were able to be sequenced due to low or degraded final RNA yield.

Differentially expressed genes (DEGs) were defined by *q* ≤ 0.05 (FDR-corrected *p* value), fold change ≥2 and overall DEGs for three comparisons are shown in Figure 3. For the first analysis, gene expression from swabs taken during the acute pharyngitis (visit 1) was compared with isolates grown in liquid culture to delineate gene expression profiles of GAS during an acute pharyngitis episode (Figure 3A; Table S4). As expected due to strong environmental changes, a large portion of the genome was differentially regulated in this comparison. Of the DEGs identified, approximately half were in the core genome (445 of 909). Many genes upregulated *in vivo* were involved in metabolic pathways such as histidine metabolism (Figures 3A and 4), similar to what was observed in a murine model of GAS necrotizing fasciitis.^34^ Fatty acid metabolism, in contrast, was downregulated in the swabs compared to growth in laboratory medium (Figure 4). A similar decrease in fatty acid metabolism was observed for Group B *Streptococcus* during colonization of the vaginal tract compared to liquid culture.^45^

To assess whether usage of the transcriptomic pan-genome and disparate *emm*-type grouping affected our results, we performed additional analyses comparing individual strains grown in liquid culture with their matched swabs. Liquid versus swab expression was assessed in four matched *emm28* strains (NC22, NC29, NC38, and NC49). As expected due to reduced power, this analysis identified fewer DEGs (211 genes) than the grouped dataset (909 DEGs) (Table S5 compared to Table S4). Despite this, concordance was very high: only 19 of the 211 DEGs in this analysis were not identified as significantly differentially expressed in the full analysis, 13 of which were in the accessory genome and 6 in the core genome. Of the top 50 most significant genes in the *emm*-grouped dataset, 43 were significant in the *emm*-type specific analysis (Table S5; Figure S7A) the remaining 7 were excluded due to low read counts and were present in the accessory genome, not the core genome. Overall, this strong agreement indicates that our bulk analysis reliably captures gene expression changes at the level of individual genetically distinct GAS isolates.

Next, we performed a comparison of GAS gene expression from all swabs obtained during an acute episode (*n* = 33, 28 NC and 5 C swabs from visit 1) and those obtained during asymptomatic carriage (*n* = 4 C swabs from visit 2). We observed differential expression of 115 genes, 93 of which were in the core genome, suggesting defined transcriptional programs in conserved genes associated with an active infection and carriage (Figure 3B; Table S6). Glucose, the preferred sugar for GAS, is depleted in the nasopharynx. GAS has been shown to use alternative sugars including mannose, commonly found on glycans in the mucosa, in these cases.^46^ We observed a mannose transport gene (*manL*) and genes involved in glucose metabolism (*pgk*, *pyk*, and *tpi*) upregulated during carriage (Figure 3B). Recent data link alternative sugar metabolism to production of bacteriocins that lead to competitive interactions within the microbiota.^46^ During carriage, GAS must persist on the mucosal surface and interact with the host microbiota, leading to cooperative and competitive interactions. We observed upregulation of a class II bacteriocin (*blp*) during carriage indicating potential complex interactions between GAS and the nasopharyngeal microbiota (Figure 3B).

As previously with our liquid versus swab analysis, we also performed matched longitudinal analysis of swabs collected at visits 1 and 2 to compare with our bulk grouped analysis. Due to the small sample size (three matched pairs), no genes reached statistical significance for differential expression. However, of the 33 most significant genes in the bulk analysis (q < 0.01), all but one were among the most significant genes in the individually matched analysis (Figure S7B; Table S7), albeit not reaching significance. This further supports that our grouping strategy preserves gene expression trends at the individual isolate level over time while providing a more robust gene set allowing for grouping of different *emm* types for transcriptional comparison.

Finally, we analyzed GAS gene expression from swabs taken during an active infection (visit 1) which would later either be cleared (non-carrier, *n* = 28, NC on visit 1) or would remain following treatment (carriage, *n* = 5, C on visit 1). These results were generally indistinguishable (Figure 3C; Table S8). When comparing these two groups, only two genes were significantly differentially expressed and, while they pass the threshold for statistical significance, expression differences were driven by one or two swabs having very high expression levels in carriers, making them unlikely to be predictive of future carriage. Our data indicate that GAS gene expression during an episode of acute inflammation in the oropharynx may not be the primary force driving GAS transition to a carrier state. A lack of significant and conserved genomic or transcriptional changes in carrier and non-carrier GAS isolates during the active pharyngitis episode as well as the epidemiology of chronic carriage in some children and lack of infection or carriage in others, indicate that external factors such as the host genetics, inflammatory environment, or host microbiota likely play a significant role in GAS infection and carriage.

## DISCUSSION

We have defined transcriptional programs across disparate human isolates using a transcriptomic pan-genome approach and determined that distinct carrier and acute transcriptional programs exist within the human oropharynx. This represents novel transcriptomic analysis of GAS obtained directly from human oropharyngeal samples. One previous study conducted in a non-human primate macaque model of long-term GAS upper respiratory tract infection delineated gene expression in GAS during the course of infection^32^ and previous studies have examined cultured GAS grown in human samples such as blood^47^ and saliva.^48^ Using a longitudinal cohort in which carriage was defined as culture positivity following antibiotic treatment with resolution of symptoms and overt inflammation, we combined whole-genome sequencing with transcriptomic profiling directly from patient throat swabs. This necessarily provides a different set of samples than swabbing asymptomatic individuals, and has been used by several studies to define GAS carriage.^18–20^ Consistent with previous epidemiological studies, isolates associated with acute infection cleared by treatment spanned multiple common *emm* types,^49–53^ whereas carriage isolates in this cohort were enriched for *emm* types 6 and 89 (Figure S1).

Genome-wide analyses did not identify a conserved genomic signature that distinguished carrier from non-carrier isolates. Although each carrier isolate contained unique amino acid polymorphisms (Table 2) not observed in acute isolates or reference genomes, these variants were heterogeneous and not shared across all carriers. Some polymorphisms occurred in genes linked to virulence or host interaction, including components of the *dlt* and *has* operons. The *dlt* operon aids in incorporating D-alanine esters into teichoic acids and modulates bacterial surface charge. Inactivation of genes in this operon could lead to changes in resistance to cationic antimicrobial peptides (AMPs). The *has* operon affects capsule production, which has been implicated to affect both carriage^41,54^ and acute infection.^55,56^ Although capsule production remained unchanged between C13–1 versus C13–2 (Figure S6A), temporally arising mutations altering capsule expression could occur during carriage. These data suggest that carriage is driven by isolate-specific genetic adaptations rather than a uniform regulatory switch. Because capsule expression influences intracellular internalization,^57^ we assessed the invasion efficiency of these strains (Figure S6B). While we observed only minor, non-significant variations between isolates C13–1 and C13–3, even subtle differences in invasion potential may facilitate intracellular persistence within the pharyngeal epithelium, serving as an evolutionary strategy to evade antibiotic clearance and establish long-term carriage. Ultimately, a larger, more comprehensive cohort of isolates is required to determine whether carriage strains exhibit a systematically distinct invasion phenotype.

Previous studies of human oropharyngeal isolates have provided important insight into GAS carriage. Whole-genome sequencing of carrier and acute infection isolates revealed similar core gene and prophage content,^58^ with limited genetic differentiation and no conserved SNPs distinguishing carriers from non-carriers.^59^ However, functional studies have shown that specific polymorphisms in key virulence regulators or surface-associated genes, including *mga*,^24^ *liaS*,^25,60^ *hasA*,^41^ and *sclA*,^26^ can reduce virulence and promote colonization in animal models. While these specific mutations were not found in our carrier isolates, this does not rule out the possibility that polymorphisms in particular genes affect the overall carriage potential of certain strains. Together, these findings and data presented here support the hypothesis that isolate-specific genetic changes in virulence-associated genes, rather than a shared genomic signature across strains, may contribute to the GAS carrier state.

We next examined transcriptional differences associated with infection state. GAS gene expression during acute infection differed markedly from growth in liquid culture (Figure 3A; Tables S4 and S5) with large numbers of DEGs including differential expression in essential core metabolic pathways (Figure 4). Although transcriptional differences will obviously exist between *in vitro* and *in vivo* environments, previous studies show that growth in traditional laboratory media sufficiently mimics oral cavity conditions, yielding similar transcriptional programs.^61^ Therefore, using standard rich laboratory medium as a transcriptional control is well justified. These baseline data will be essential for future laboratory experiments evaluating gene importance during acute oropharyngeal GAS infections and assessing future GAS vaccine candidates. In contrast, we observed no major transcriptional differences during acute infection between isolates that would later resolve versus those that would persist as carriers, suggesting that gene expression during acute pharyngitis is not predictive of future treatment failure or long-term carriage (Figure 3C; Table S8).

Importantly, transcriptomics revealed bacterial gene expression changes when comparing GAS during an acute infection (visit 1) to those remaining post-treatment (visit 2) (Figures 3B and S7B; Tables S6 and S7), likely reflecting changes in response to the inflammatory environment and microbial community of the throat. The only published transcriptional data from GAS bacteria during a *in vivo* “carriage-like” condition comes from an animal model of long term upper respiratory tract infection in macaques over a duration of several weeks.^32^ In this study, temporal changes in GAS gene expression were examined over the course of the infection. The acute phase of infection was defined as days 4–23 following intranasal inoculation when acute infection indictors like tonsilitis and pharyngitis score, and C reactive protein levels, were high. The asymptomatic or carriage phase was defined as days 23–58 when these infection markers were low but GAS was still present. Both GAS gene expression^32^ and host responses,^62^ were evaluated. Comparison of our data with this study reveals partial overlap in genes associated with acute infection and asymptomatic phases, but also notable differences. Some of the most highly down-regulated genes observed in the macaque study on day 32 (asymptomatic infection) versus day 16 (acute infection)^33^ include *hlyX* (spy0317), *atpF* (spy0577), *proB* (spy1372), and *accA*, (spy1485), among others. While these genes were also all down-regulated in our asymptomatic carrier conditions, only one of them, *accA*, reached significance. Some of the most highly up-regulated genes in the day 32 samples compared to day 16 included *adrC* (spy 0078), *fusA* (spy0232), *tcyA* (spy0982), and *hup* (spy1223). *hup* and *fusA* were also significantly up-regulated in our dataset during carriage but *adrC* and *tcyA* were not significantly differentially expressed.

Interestingly, some factors commonly associated with GAS virulence and inflammation such as enterotoxins, were not found to be significantly up-regulated during an active infection when compared to the carriage state in our swabs. In macaques, GAS genes such as *speB (spy1735)*, *smeZ (spy1702)*, *and scpA (spy1715)* were significantly upregulated during the first acute phase of infection.^32^ In our dataset, no difference was seen in the expression of these genes comparing the visit 1 and visit 2 swabs. Other genes identified in that dataset as being correlated with acute infection such as *covS* and *sic* were up-regulated in our dataset but not significantly. Collectively, these analyses indicate that although certain transcriptional patterns are conserved between experimental and natural carriage, key differences may highlight the context-specific nature of GAS gene regulation in various models.

### Limitations of the study

Our study has limitations, including strain heterogeneity, modest sample size, and the *in vitro* conditions of the biofilm assay, potentially limiting *in vivo* applicability. Importantly, we are also not able to disentangle bacterial adaptation from antibiotic- or microbiota-driven effects on samples from visit 2. The genomic data also stems from individual isolates from a patient. While we see almost complete genome conservation in patients with our compared longitudinal isolates, this does not preclude the possibility of intra-subject heterogeneity which was not captured in this analysis. While we have a broad range of isolates more representative of the diversity of strains in the population, it also makes transcriptomic differential expression comparison more difficult as the strains being compared are not genetically identical, as in the macaque study. This creates the need for a transcriptional pan-genome to examine overall gene expression profiles. A primary limitation of pan-genome analysis is that transcriptional data from the accessory genome can be difficult to interpret across diverse serotypes. While core genome expression provides a more straightforward comparison between strains, analyzing the accessory genome remains vital. Even if direct comparisons are complex, this expression data still offers a snapshot of the biological processes occurring during acute infection and carriage in diverse strains. Some accessory genes are found in particular *emm* types more than others and there is an over-representation of *emm* type 6 in our carriage isolates, this leads to skewed statistics showing possible over-expression of *emm-*type specific genes during carriage. We have focused much of the discussion on genes found in the core genome in most of the isolates, but care should be taken when interpreting the expression patterns of *emm-*type specific genes in the dataset. As such, genes in the accessory genome rather than the core pan-genome are noted in Table S3 and delineated in the subsequent analysis tables.

Despite these limitations, our study reports GAS transcriptomic data from a human cohort using oropharyngeal swabs. We demonstrate that neither conserved genomic variations nor acute-phase transcriptional profiles are able to reliably predict GAS carriage in humans. Instead, carriage may arise from a complex interplay between isolate-specific genetic adaptations and host or microbial ecological factors. These findings establish a foundation for future studies integrating host responses and microbiome dynamics to define the determinants of GAS persistence.

### RESOURCE AVAILABILITY

#### Lead contact

- Requests for further information and resources should be directed to and will be fulfilled by the lead contact, Laura C. Cook (lcook@binghamton.edu).

#### Materials availability

- This study did not generate unique reagents.

#### Data and code availability

- RNA-seq data have been deposited in the NCBI Gene Expression Omnibus (GEO) database under accession number GSE309825 and are publicly available as of the date of publication.
- Whole genome sequencing data are available in the NCBI Sequence Read Archive (SRA) database under Bioproject PRJNA1357344 for fastq files. NC are labeled as Acute with the accompanying patient number. Individual accession numbers are provided in Table 1.
- This paper does not report original code.
- Any additional information required to reanalyze the data in this paper is available from the lead contact upon request.

## STAR★METHODS

### EXPERIMENTAL MODEL AND STUDY PARTICIPANT DETAILS

#### Human participants

- Age/Developmental Stage: Children aged 5–15 years old.
- Sex/Gender: Both male and female children were enrolled. Detailed demographic breakdowns for sex, gender, ancestry, race, and ethnicity were not collected or reported in this study.
- Inclusion/Exclusion Criteria: Children presenting with acute pharyngitis who tested positive by rapid antigen detection test (RADT) at four pediatric clinics in Madison, WI, USA, were eligible for inclusion. Key exclusion criteria included antibiotic use within the preceding 30 days, known allergies to *β*-lactam antibiotics, or treatment with a non-*β*-lactam antibiotic.
- Ethics and Consent: All procedures were conducted in accordance with protocol #2015–1456-CP016 approved by the Institutional Review Board (IRB) at the University of Wisconsin. Written informed consent was obtained from parents/guardians, and assent was obtained from all participating children prior to enrollment.
- Infection Classification & Sampling: Enrolled participants received standard 10-day courses of amoxicillin or penicillin V and were evaluated at follow-up (days 13–14 post-treatment) via posterior pharyngeal swab cultures. Participants were classified as “non-carriers” if follow-up GAS cultures were negative, or as “probable carriers” if cultures remained positive. Probable carriers were re-evaluated ~13–14 days later; persistent GAS culture positivity confirmed “carrier” status.
- Genotypic Verification: Paired GAS isolates from initial presentation and final follow-up were confirmed to match by *emm* typing via PCR. Differential expression analyses were restricted to participants with concordant *emm* type paired isolates.

#### Microbe strains

- Species/Strains: Group A *Streptococcus* (GAS; *Streptococcus pyogenes*) clinical isolates.
- Source & Collection: Primary clinical isolates were obtained directly from posterior pharyngeal swab cultures of pediatric participants presenting with acute pharyngitis and/or persistent carriage in Madison, WI, USA.
- Genotype: Isolate genotypes were characterized by PCR-based *emm* typing. Matched isolates from initial and follow-up visits demonstrating concordant *emm* types were selected for downstream genomic sequencing and transcriptomic profiling.
- Storage & Maintenance: Recovered isolates were preserved and stored at −80°C until use.
- Strain Identification: Comprehensive metadata for all bacterial isolates used in this study—including strain designations, *emm* types, subject associations, and sequencing status—are listed in Table 1.

#### Cell lines

- Cell Line Name: Detroit 562 (ATCC CCL-138). ATCC verifies all cell lines using STR profiling and tests for *Mycoplasm* routinely.
- Species & Sex: *Homo sapiens* (Human), female.
- Cell Type/Tissue Origin: Continuous human pharyngeal carcinoma epithelial cell line derived from a metastatic pleural effusion.
- Culture Maintenance & Care: Cells were cultured in minimum essential medium eagle 1X with glutamine (MEM, Corning) and 10% fetal bovine serum (FBS, Gibco), and maintained at 37°C in a humidified atmosphere containing 5% CO_2_. Cells were passaged upon reaching 80–90% confluence using trypsin-EDTA. Cells from ATCC are passaged once and split before storage and were only passaged a maximum of 10 times prior to use.
- Experimental Application: Used as an *in vitro* human pharyngeal epithelial model to evaluate GAS host cell invasion.

### METHOD DETAILS

#### Swab collection

Children were recruited from four pediatric clinics in Madison, WI, USA. Participants were eligible if they were between 5 and 15 years old, had a diagnosis of acute pharyngitis, and had not received antibiotics in the preceding 30 days. Children were excluded if they had an allergy to β-lactam antibiotics or if they were treated with an antibiotic other than a β-lactam. At presentation, a swab was collected from the posterior pharynx (tonsillar pillars and posterior pharyngeal wall) and tested using a rapid antigen detection test (RADT). If the RADT was positive, the child and family were informed of study eligibility. Written informed consent and assent were obtained prior to enrollment. Following enrollment, two additional posterior pharyngeal swabs were collected. One swab was sent to the laboratory for culture of GAS using standard methods and the resulting cultured isolate was preserved. The second swab was immediately stored at −80°C for future transcriptional profiling. Participants were then treated with either amoxicillin or penicillin V for 10 days at the discretion of the provider. Participants returned for follow-up 13–14 days after initiation of treatment. If the child was asymptomatic and showed no signs of pharyngeal or tonsillar inflammation, a double swab of the posterior pharynx was collected. As on the first visit, one swab was sent to the laboratory to be cultured for GAS and the cultured isolate was preserved. The second swab was stored immediately at −80°C. If the follow-up swab was negative for GAS on culture, the patient was deemed to have presented with an acute infection and labeled a “non-carrier”. If the follow-up swab was positive, the child was classified as a probable carrier. Probable carriers returned approximately 13–14 days later and an additional set of swabs was obtained and treated as on previous visits. If the final cultured swab was still positive for GAS, the subject was deemed a confirmed-carrier and the swab and cultured isolates were stored at −80°C. Cultured individual isolates from a given carrier patient were tested by PCR to demonstrate the same *emm* type in the first and last swabs. Only those with concordant *emm* type were compared for differential gene expression. The initial study design tested for concordant *emm* types for return isolates but did not save the isolates. Matched isolates were sequenced as available and are listed in Table 1. Comparisons of symptom prevalence between carrier and non-carrier groups were performed using Fisher’s exact test for each symptom. All *p*-values are nominal, and statistical significance was defined as *p* < 0.05. Swabs were collected under protocol #2015–1456-CP016 approved by the University of Wisconsin Institutional Review Board (IRB).

#### emm typing

*emm* typing of isolates was done as previously published.^38,63^ Genomic DNA from isolated cells was amplified using published primers *emm* Sense: TATTGGCTTAGAAAATTAA and *emm* Antisense: GCAAGTTCTTCAGCTTGTTT.^38,63^ PCR amplified DNA was sequenced and sequences aligned to the closest *emm* type using NCBI Blast. The nearest sequence match was analyzed for SNPs in the first 180 residues and *emm* types were identified if no SNPs were found.

#### Biofilm formation

Biofilm assays were done as previously described^64,65^ with minor modifications. Bacterial cultures were grown overnight in Todd Hewitt medium +2% yeast extract (THY) statically at 37°C. Cultures were diluted to an OD_600_ of 0.05 in fresh THY +0.5% glucose and 1 mL of the cell suspension was added to wells of a 24 well plate in at least technical triplicate and incubated at 37°C. After 24 h, the culture medium was removed, and the wells were washed 3 times with 1 mL phosphate buffered saline (PBS) to remove unattached cells. 1 mL of 0.1% crystal violet solution was added to each well and incubated at room temperature for 10 min. Excess crystal violet was removed, and the wells washed another 3 times with 1 mL PBS. Remaining stain was dissolved in 1 mL 95% ethanol and absorbance at OD_590_ was measured. The average of ≥2 technical replicates was counted as a single point and the experiment was performed for each strain in at least biological triplicate. The lab strain NZ131 was included on each plate as an internal control for variability and values are shown relative to the NZ131 from the same plate. Values shown are the average and standard deviation of the biological replicates.

#### Capsule production

Capsule production was measured as previously described^66^ with modifications. Overnight bacterial cultures grown in THY were diluted 1:20 in 10 mL fresh medium and grown to OD_600_ of 0.3–0.5, pelleted and washed with 10 mL of 10 mM Tris (pH 7.5). The wash was repeated and the pellets were resuspended in 1.5 mL H_2_O and pelleted again. Final pellets were resuspended in 0.5 mL H_2_O. 100 μL was removed and plated on THA plates for CFU/mL counts. 1 mL of CHCl3 was then added to the culture and vortexed for 60 s. The cells were then incubated at room temperature for 10 min on an orbital shaker and pelleted. 100 μL of the aqueous phase was then added to duplicate wells of a 96 well plate. 100 μL of hyaluronic acid (HA, Fisher Sci 50–186–9545) standards (containing 0, 0.1, 0.5, 1, 5, and 10 μg diluted in PBS were added in duplicate to the plate. 200 μL Stains-all solution (9 mL formamide, 9 mL H_2_O, 10 μL acetic acid, 180 μL Stains-all (Fisher Sci AAJ6263003)) was added to each well and mixed. The plate was incubated at room temperature in the dark for 10 min and the absorbance OD_640_ read. Capsule measurements were determined by extrapolating the HA standard curve and expressed as femtograms/CFU. Values shown are mean ± SD and the result of ≥3 biological replicates per isolate.

#### Invasion assay

Streptococcal invasion of human pharyngeal epithelial cells (Detroit 562) monolayers in 24-well plates was performed as previously described with slight modification.^67,68^ Bacterial cultures were diluted 1:10 in fresh medium and grown to logarithmic growth phase (OD600 = 0.4). Following growth to an OD_600_ of 0.4, bacterial cells were pelleted by centrifugation, with the volume needed calculated as 0.4/OD_600_ = volume to centrifuge. The pellet was washed once with 1 mL of antibiotic-free culture medium, minimum essential medium eagle 1X with glutamine (MEM, Corning) and 10% fetal bovine serum (FBS, Gibco), and resuspended in 1 mL of the same medium. Concurrently, the culture medium on the cell monolayers of the 24-well plates were removed, and each well was washed once with 500 μL of PBS. To achieve a target multiplicity of infection (MOI) of approximately 100:1 (bacteria: cell), the bacterial suspension was diluted in antibiotic-free medium based on the epithelial cell count per well. For each sample, at least two wells were inoculated with 500 μL of the prepared bacterial inoculum. The plates were incubated for four hours at 37°C with 5% CO2. During this incubation period, serial 10-fold dilutions of the initial inoculum were prepared and drop plated to determine initial bacterial counts. Following the adherence and invasion period, the monolayers were washed twice with PBS to remove unattached bacteria. 500 μL of fresh medium supplemented with gentamicin (100 μg/mL) was added to each well to kill extracellular bacteria and incubated for 1 h at 37°C with 5% CO2. Following the incubation, the wells were washed three times with PBS. The cells were detached and lysed by the addition of 500 μL of 0.05% trypsin-EDTA +0.25% Triton X-100 per well and incubated at 37°C for 5–10 min. Intracellular bacteria were collected by vigorously pipetting up and down and the lysates were collected. Serial dilutions of the lysate mixture were plated, and total colony-forming units were quantified following overnight incubation.

#### Minimum inhibitory concentration tests

Minimum inhibitory concentration (MIC) assays were done using MIC Test Strips (MTS, Liofilchem) for clindamycin (0.016–256 μg/mL, 92072), amoxicillin (0.016–256 μg/mL, 92021), penicillin G (0.002–32 μg/mL, 92103), and erythromycin (0.016–256 μg/mL, 92051). Frozen culture stocks were streaked onto Blood Agar Plates (BAP, Hardy Diagnostic, A10). Individual β hemolytic colonies were grown overnight in THY. Per manufacturer’s instructions, overnight cultures were diluted to OD_600_ = 0.5 and sterile swabs were used to streak lawns of bacteria on BAPs. Antibiotic strips were added, and the plates were incubated for the manufacturers’ recommended time and temperature. MIC values were recorded per manufacturer’s instructions. Resistance to antibiotics were defined as follows: penicillin MIC ≥4 mg/L, amoxycillin MIC ≥1 mg/L, clindamycin MIC ≥1 mg/L, erythromycin MIC ≥1 mg/L.

#### Preparation of DNA for Illumina short-read whole genome sequencing (WGS)

Genomic DNA was prepared using overnight cultures of GAS grown in THY at 37°C statically. Cultures were pelleted and pellets were resuspended in 150 μL lysis buffer (10 mM Tris, 1 mM EDTA, pH 8.0 with 1% w/v SDS) and incubated at 55°C for 15 min 1/5 volume 3 M NaAc was added and the cells were incubated at 65°C for 30 min and then incubated on ice for 1 h. Samples were centrifuged at 15,000 ×g for 15 min. DNA in the supernatant was precipitated by adding 2 volumes of ice-cold 100% ethanol followed by incubation at −80°C for at least 60 min. DNA was pelleted by centrifugation at 15,000 ×g for 10 min. The supernatant was removed and the pellets were air dried. The resulting DNA pellet was resuspended in 100 μL water and the concentration and purity measured on a Nanodrop OneC (Thermo Fisher). DNA libraries for WGS were prepared from 500 ng of gDNA in-house using the Illumina Nextera Flex DNA library preparation kit (Illumina 20018704) per manufacturer’s instructions using the Nextera DNA CD Indexes (ThermoFisher 20018707). Final libraries were sent to the University of Illinois at Chicago Research Resources Center for quality control, sequencing, and analysis.

#### Preparation of DNA for PacBio long-read whole genome sequencing

High molecular weight (HMW) DNA was prepared for Pac Bio sequencing libraries using the HMW DNA Extraction kit for Tissue and Bacteria (NEB T3060) according to manufacturer’s instructions for low input Gram positive bacterial samples with some modifications. Overnight cultures grown in THY were spun down to an equivalent of OD_600_ = 4.0. Pellets were resuspended in 150uL of STET buffer (10mM Tris-HCl, 1mM EDTA, 100mM NaCl and 5% Triton X-100) with freshly added lysozyme at 50 mg/mL. Homogenization was carried out according to kit manual, using the thermal mixer protocol at 1400 rpm. DNA was sheared using the Covaris g tubes (Covaris 520079) per manufacturer’s instructions using 100 μL/sample and spinning at 9400 rpm for 2 min on each side. Sheared DNA was then cleaned up using the 0.45X AMPure PB beads (PacBio 100–265–900) and Elution Buffer (PacBio 101–633–500) following the PacBio protocol. The quality and quantity of DNA was checked using a Nanodrop OneC and only samples with A260/280 ratios of >1.8 were used to make libraries. PacBio libraries were made in-house with the SMRTbell Express Template Prep Kit 2.0 (PacBio 100–938–900) using the SMRTbell Barcoded Adapter Plate 3.0 (PacBio 102–099–200) per manufacturer’s instructions. Libraries were sent to the University of Illinois at Chicago (UIC) Genomics Core facilities for quality control and sequencing on a Sequel II SMRT Cell.

#### Genome assembly

Prior to assembly, PacBio data were filtered to remove reads shorter than 1000 bp and trimmed to remove adapters using Porechop v0.2.3.^69^ Filtered and trimmed reads from each isolate were individually assembled using Flye v2.9^70^ with the PacBio HiFi option and command line options genome-size = 2m and asm-coverage = 100. Illumina reads sequenced from the same isolate were aligned to assembled contigs using the Burrows-Wheeler Aligner MEM algorithm (BWA MEM) v0.7.17 and the mpileup output from the alignment was processed to generate a polished assembly for each isolate.^71^ Each polished assembly was used for further alignment and polishing, repeating until no changes could be detected. Prokka^72^ was used to detect open reading frames (ORFs) and perform functional annotation of the final polished assembly for each isolate.

#### Isolate pan-genome analysis

Assembled genomes and functional annotations for each sequenced GAS isolate were loaded into Anvi‘o^73^ alongside reference genomes HG316453.2, NC_002737.2, NC_006086.1, NC_008021.1, and NC_008024.1 using the anvi-gen-contigs-database, anvi-import-functions, and anvi-gen-genomes-storage programs. Additional functional annotations were added for each genome using the anvi-scan-trnas, anvi-run-hmms, anvi-run-ncbi-cogs, and anvi-run-kegg-kofams programs to add tRNA, HMM, COG, and KOfam information to the analysis. Single-copy core genes (SCG) were identified for each genome using the anvi-run-scg-taxonomy program. The pan-genome analysis was run on all isolate and reference genomes using the anvi-pan-genome program. Average nucleotide identity was computed using the anvi-compute-genome-similarity program with the pyANI option.^74^ SCG were extracted from the pan-genome analysis, concatenated and aligned using the anvi-get-sequences-for-gene-clusters program for subsequent multi-locus phylogenetic analysis. Amino acid positions with gaps in >50% of genomes were excised from the multi-locus alignment using trimAl.^75^ FastTree^76^ was used to infer the phylogenetic relationship from the multi-locus sequence alignment and was loaded into Anvi‘o using the anvi-import-misc-data program. A figure was generated using the anvi-display-pan program to display ORFs, ANI, and the inferred phylogeny, with colors added manually to each track to highlight the *emm* types for each genome. To gain a better understanding of the functional differences between the SCG and the remaining set of genes in the isolate pan-genome, termed here as the “accessory genome”, a pie chart was generated from the COG20 categories annotated for each set of gene clusters using the R library ggplot2.^77^

To examine an apparent discordance in phylogenetic placement of NC-37 (*emm*1) as part of the *emm*89 clade, and C7 (*emm*89) as part of the *emm*1 clade by the concatenated SCG tree, each SCG gene cluster was individually examined. Each of the 1,203 SCG gene clusters were exported, aligned with MUSCLE v5,^78^ trimmed with trimAl (gap threshold 0.5), and a maximum-likelihood tree was inferred with FastTree. Robinson-Foulds (RF) distances between each per-gene tree and the SCG tree were computed in R using the ape^79^ and phangorn^80^ packages. Polytomies in per-gene trees, common for short conserved genes, were randomly resolved with multi2di prior to RF computation. The five nearest phylogenetic neighbors of NC-37 and C7 in each per-gene tree were identified by patristic distance using cophenetic.phylo. For each of the 1,203 per-gene SCG trees, the five nearest phylogenetic neighbors of NC-37 and C7 were recorded and bar plots were generated from the counts using R with the ggplot2 package. The genomic coordinates of all genes that were concordant or discordant with the *emm* classification for either isolate were examined to see if they were collocated within a smaller region of the respective genome, but no evidence of clustering was found.

To assess whether the anomalous phylogenetic placements of NC-37 and C7 could be attributed to chimeric assemblies derived from mixed-culture samples, raw Illumina short-read and PacBio HiFi long-read sequences for each isolate were mapped against three references, the isolate’s own assembly, the *emm*1 reference genome (NC_002737), and the *emm*89 reference genome (HG316453). Mapping and variant calling were performed using Snippy,^81^ which employs BWA for short-read alignment, minimap2 for long-read alignment, and FreeBayes^82^ for haploid variant calling. Positions with allele frequencies between 5% and 95% were considered evidence of a mixed population; no such positions were identified across any of the twelve comparisons, ruling out mixed-culture contamination as an explanation for the observed discordance. To validate the FastTree phylogeny and rule out tree-inference artifacts as an explanation for the anomalous placement of NC-37, the concatenated SCG alignment was re-analyzed using IQ-TREE 2^83^ with automatic substitution model selection via ModelFinder and 1,000 ultrafast bootstrap replicates. The best-fit model selected by the Bayesian information criterion was Q.mammal+F+I + G4. The resulting topology was highly concordant with the FastTree phylogeny, with bootstrap support of 99–100 at the branches placing NC-37 within the *emm89* clade despite *emm*-type prediction.

#### RNA extraction and depletion of eukaryotic and ribosomal rRNA

For liquid samples, overnight cultures of GAS strains were grown in THY medium. In the morning, cultures were diluted 1:10 in fresh THY medium and grown at 37°C static until the OD600 reached 0.4–0.6. Cells were then spun down and resuspended in 500 μL DNA/RNA Shield (Zymo) before storage at −80°C until extraction. When ready, samples were thawed at room temperature, centrifuged to pellet the cells and the DNA/RNA Shield removed. Pellets were then resuspended in 200 μL lysis solution (TRIzol Max Bacterial Enhancement Reagent (ThermoFisher 16122012) which had been pre-heated to 95°C mixed with 1 mg/mL lysozyme and 50 U/mL mutanolysin) and moved to a MagMAX prime bead beating tube (ThermoFisher A58155). For swabs, 500 μL DNA/RNA Shield was added to frozen swabs and then vortexed for 30–60 s and then the tubes containing the swabs were then sonicated in a sonicator bath for 5 min to help with removing adherent cells. Following sonication, swabs were again vortexed for 30–60 s and then the tubes were spun down for 5 min at 4°C. The supernatant and swab were removed and the resulting pellet resuspended in 200 μL lysis solution and moved to MagMAX bead beating tubes as above.

All samples were placed in an OMNI Bead Ruptor 12 (Omni International, 19–050A) and beat twice for 4 min each with a 30 s break in between. The mixtures were then incubated at 37°C for 30 min to allow additional enzymatic lysis and then incubated at 95°C for 5 min. RNA was then collected using the TRIzol Max Bacterial RNA Isolation kit (ThermoFisher 16096–040) per manufacturer’s instructions including the phase separation and RNA precipitation steps. RNA pellets were air dried and resuspended in 45 μL DEPC-treated water. The Turbo DNA-free kit (ThermoFisher AM1907) was used per manufacturer’s instructions to remove any remaining DNA in the sample. The final RNA quantity and quality were measured on a NanoDrop OneC.

Depletion of eurkaryotic RNA was done on the RNA from the swab samples using the MICROEnrich kit (ThermoFisher AM1901) per manufacturer’s instructions. The final precipitation step was skipped and the samples were taken immediately into the MICROBExpress kit protocol (ThermoFisher AM1905) per manufacturer’s instructions to remove bacterial rRNA. For liquid cultures, bacterial rRNA was removed using the same MICROBExpress kit using 2–10 μg starting RNA in ≤15 μL total volume.

#### RNA-sequencing library preparation and sequencing

RNA-seq libraries were prepared in house using two different protocols. Libraries from swabs processed before 2020 were prepared using the Kapa Stranded RNASeq Library Preparation kit (KK8400) per manufacturers’ instructions for purified mRNA with input range of 10–100 ng total. RNA was fragmented at 94°C for 6 min with random primers and separate Illumina adapters were ligated onto each samples. The libraries were then frozen at −20°C and sent to the University of Illinois at Chicago Genomics facility for Tapestation QC and Quibit quantification prior to sequencing on a HiSeq 2500. Libraries from swabs processed after 2020 were made using the NEB Next Ultra II Directional RNA library prep kit (NEB E7765) per the manufacturer’s protocol for purified mRNA. mRNA input amounts ranged from 1–100 ng total, in line with the recommended range for the library preparation kit. Briefly, rRNA depleted RNA was first fragmented at 94°C for 10 min with random primers. First and then second strand cDNA was synthesized, followed by cDNA purification using SPRIselect beads. Ends were prepped and NEBNext adaptors were ligated to cDNA strands. Samples were again purified using SPRIselect beads, followed by PCR enrichment of adaptor ligated DNA using unique indexed forward and reverse primers. Samples were purified once more using SPRIselect beads, and library quality was analyzed using a NanoDrop (Thermo). The libraries were then frozen at −20°C and sent to the University of Illinois at Chicago Genomics facility for Tapestation QC and Quibit quantification prior to sequencing on an Illumina NovaSeq X 10B, 2×150bp reads.

#### RNA-seq quantification and differential analysis

A custom FASTA database (called our transcriptional pan genome) was generated containing all of the coding sequences (CDS) from reference genome GAS ATCC 12344 and CDS from HG316453.2, NC_002737.2, NC_006086.1, NC_007296.2, NC_008021.1, NC_008022.1, and NC_008024.1 that could not be found in the ATCC strain using BWA MEM v0.7.17.^71^ Raw RNA-seq reads were aligned to this custom database using BWA MEM. Percent PCR duplication was assayed using Picard MarkDuplicates, but no pruning of data was performed for subsequent steps (Picard Toolkit. 2019. Broad Institute, GitHub Repository. https://broadinstitute.github.io/picard/; Broad Institute). The expression level of gene features (CDS regions from the reference) were quantitated separately for each sample using FeatureCounts v2.0.3^84^ as raw read counts of the stranded libraries. Differential analysis of quantitated gene features as compared with patient group (non-carrier vs. carrier) or sampling method (liquid vs. swab) was performed using the software package edgeR^85,86^ on raw sequence counts using each individual biological sample as a replicate observation in the statistics. Additional differential expression analyses were separately run on non-carrier (*n* = 4 matched by *emm* type) and carrier (*n* = 3 matched longitudinally) samples with a repeated measures design where samples from the same individual were processed using both liquid and swab methods. Prior to analysis, the data were filtered to remove any features that had less than 5 total counts summed across all samples. Normalization of gene expression was performed using a global normalization strategy, in line with standard best-practices for RNA-seq: normalized expression was computed as counts per million (CPM) and TMM (trimmed mean of M-values) factors were computed using edgeR to account for outlier effects on normalization. We measured robust expression of genes across the genome in swab samples: out of 2,365 total genes measured from 58 libraries, on average we obtained expression from 861.4 genes ±60.2 (mean and standard error) with at least 10 counts. Statistical tests were performed using the “exactTest” function in edgeR. Adjusted *p*-values (q-values) were calculated using the Benjamini-Hochberg false discovery rate (FDR) correction. Significant gene features were determined based on an FDR threshold of 0.05 (5%). Genes expressed in at least 1 library were compared against the NCBI COG20 database using DIAMOND^87^ and split into SCG and accessory genome subsets based on comparison with the Anvi’o gene clusters. A pie chart was generated from the COG20 categories annotated for each set of genes using the R library ggplot2.

### QUANTIFICATION AND STATISTICAL ANALYSIS

Statistical analyses are described in the figure legends or in more detail in the method details section. Fisher’s exact test was used to determine statistical significance for *emm* distributions. Students *t* test was used to determine differences between carrier and non-carrier biofilm formation. Paired *t* test was used to determine differences between specific isolates in capsule and invasion assays. **p value <0.01 was considered significant. *N* values are listed in individual figure and table legends.

## Supplementary Material

1

2

4

5

6

7

8

9

10

11

SUPPLEMENTAL INFORMATION

Supplemental information can be found online at https://doi.org/10.1016/j.celrep.2026.117925.

## Figures and Tables

**Figure 1.**
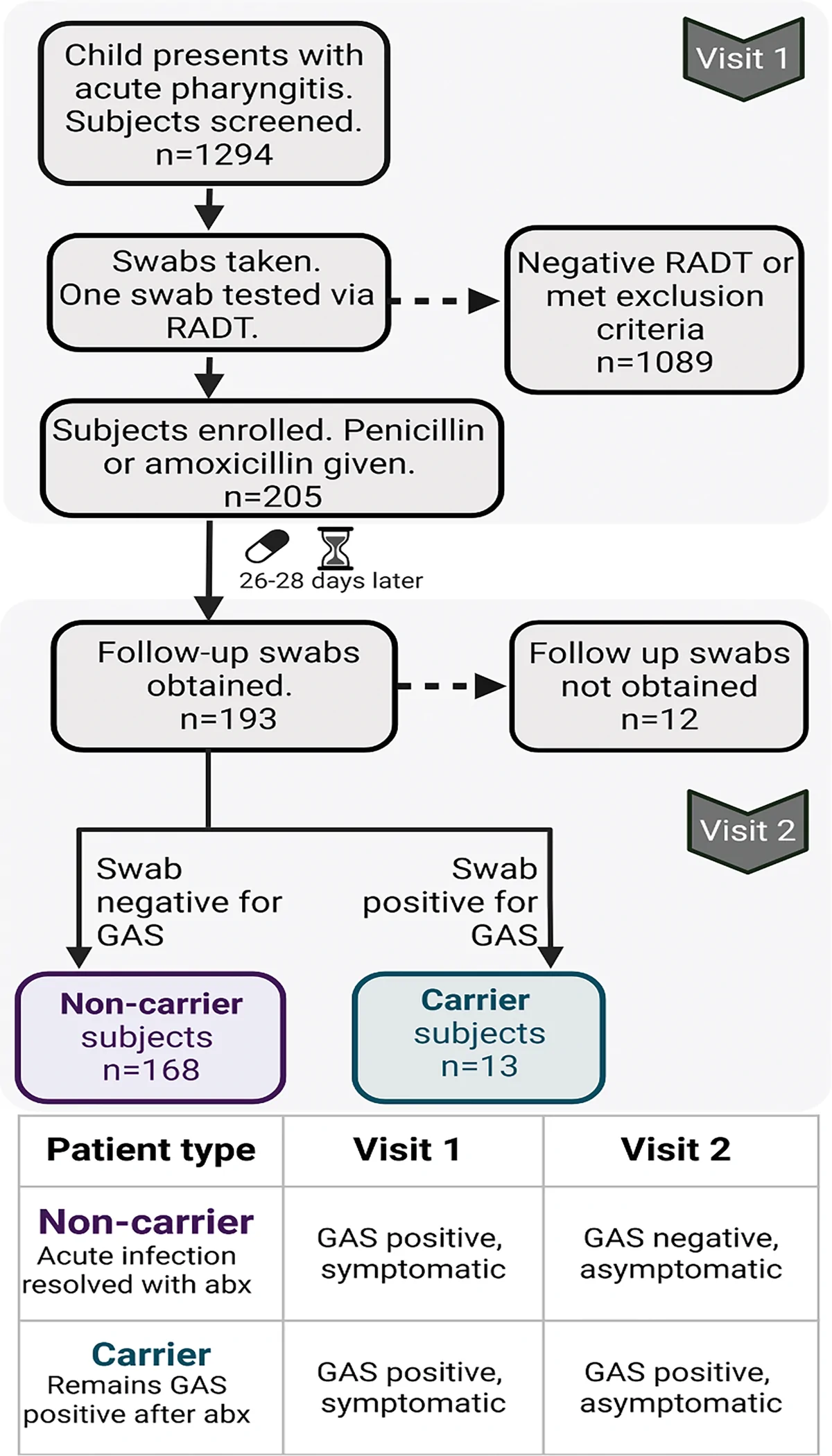
Sample collection diagram outlining the patient swab collection protocol Flowchart of study participants and swab sample collection. Eligible participants were between 5–15 years of age, not allergic to β-lactam antibiotics, and positive for GAS using a rapid antigen detection test (RADT). Swabs are collected during the initial visit (visit 1) and after a course of β-lactam antibiotic (penicillin or amoxicillin) treatment (visit 2). Patients with a positive throat culture for GAS 26–28 days after visit 1 were considered GAS carriers and isolates were identified as carriage isolates (C). Patients who are negative on GAS throat culture at visit 2 are assumed to have had an acute infection appropriately treated with antibiotics and termed non-carrier (NC). Figure created in Biorender.

**Figure 2.**
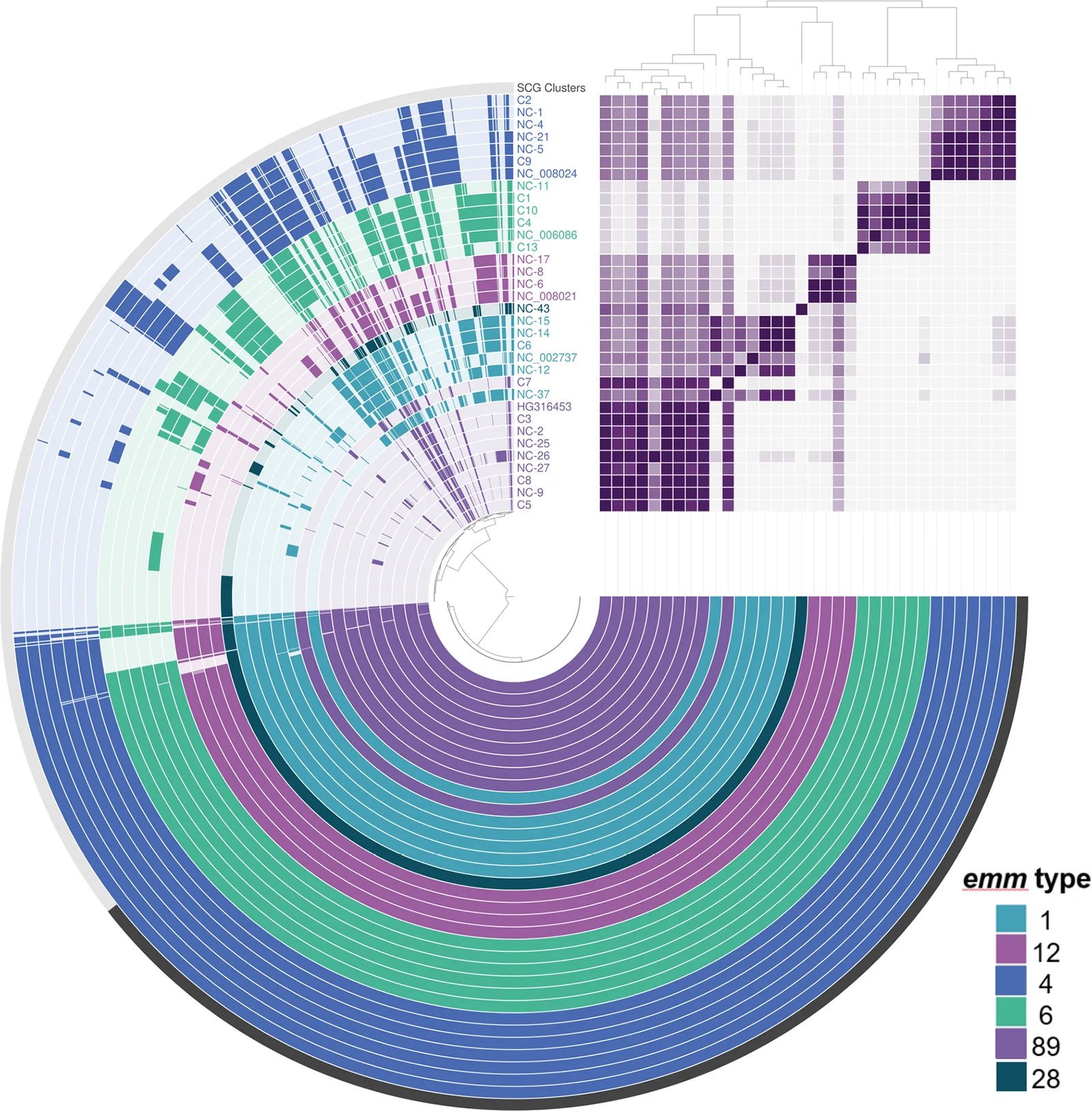
Anvi’o pan-genome analysis of *Streptococcus pyogenes* isolates Circular representation of the pan-genome generated using Anvi’o. Concentric rings correspond to individual GAS genomes, including reference genomes HG316453.2, NC_002737.2, NC_006086.1, NC_008021.1, and NC_008024.1, with colors indicating *emm* type (legend, bottom right). Radial bars show the distribution of open reading frames (ORFs) across gene clusters identified in the pan-genome analysis. The dendrogram reflects the phylogenetic relationships inferred from concatenated single-copy core gene (SCG) alignments. The heat map (top right) depicts pairwise average nucleotide identity (ANI) calculated using the pyANI method, with darker shading indicating higher ANI values.

**Figure 3.**
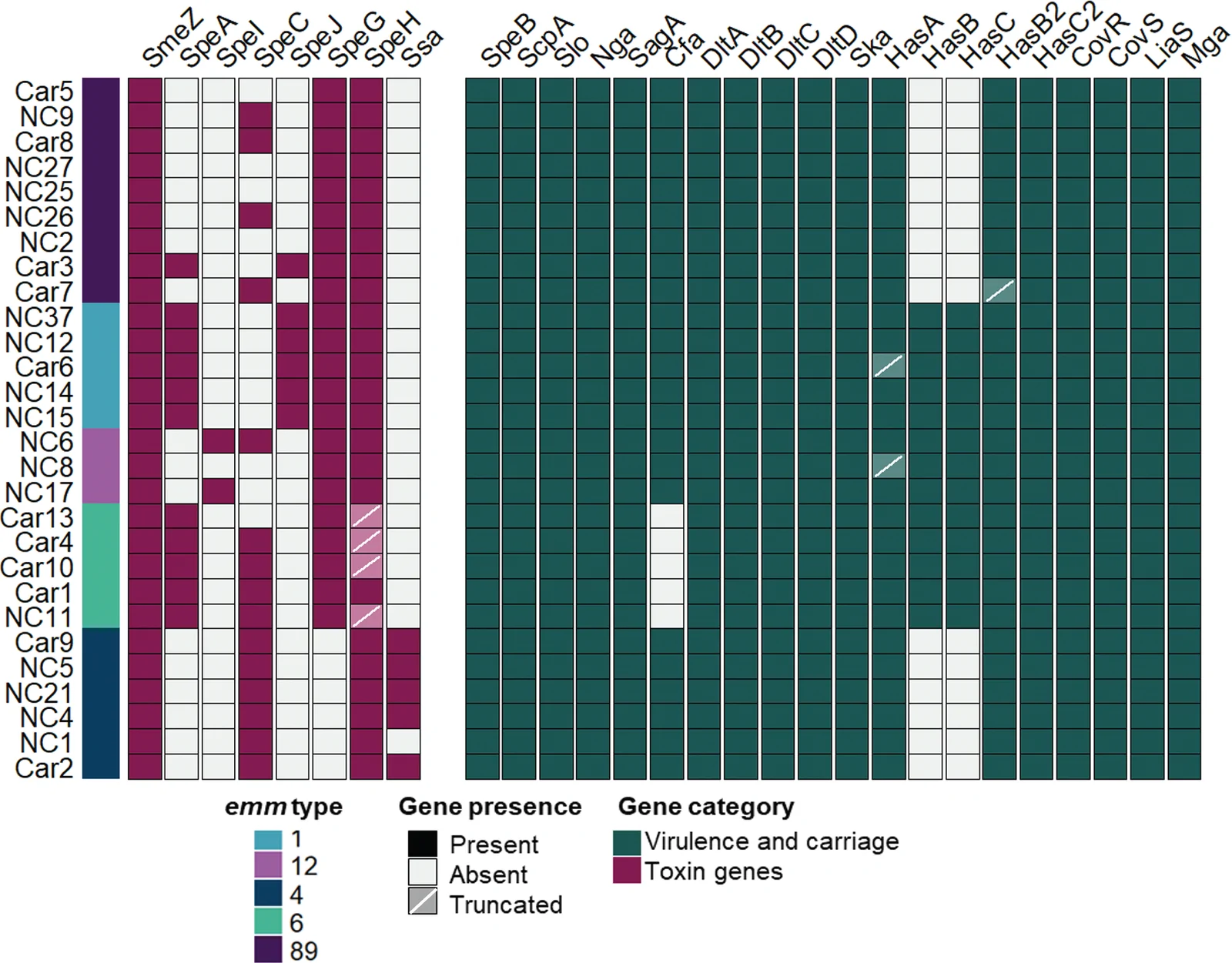
Distribution of virulence- and toxin-associated genes across *Streptococcus pyogenes* isolates Heat map showing the presence, absence or truncation of selected genes across sequenced GAS isolates. Rows correspond to individual isolates, grouped by *emm* type (left color bar) with carrier (Car) and non-carrier (NC) isolates indicated. Columns represent protein alleles, with toxins (dark pink) and virulence/carriage-associated factors (dark green) indicated above. Protein status is shown as present (dark), absent (white), or truncated (medium), based on comparison with reference protein sequences. This analysis highlights protein content differences among isolates generally groups by *emm* types rather than phenotypic category.

**Figure 4.**
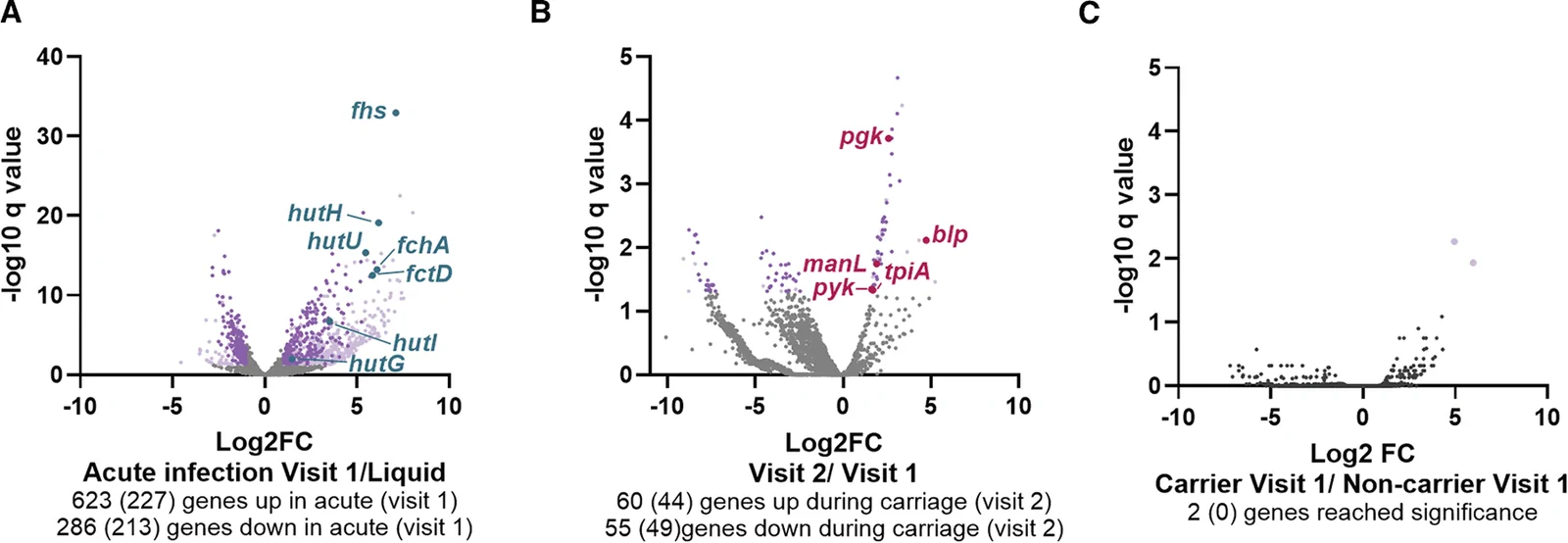
Differential gene expression across infection state, sampling condition and treatment outcome in *Streptococcus pyogenes* Volcano plots showing pairwise comparisons of GAS gene expression. (A) Throat swabs collected during acute infection (visit 1, *n* = 33) compared with growth of isolates in liquid culture (*n* = 21). (B) Acute infection (visit 1, *n* = 33) compared with asymptomatic carriage following antibiotic treatment (visit 2, *n* = 4). (C) Acute infection that resolved (non-carrier patients, visit 1, *n* = =28) compared with acute infection that did not resolve following antibiotic treatment (carrier patients, visit 1, *n* = 5). Each point represents a gene, plotted by log_2_ fold change and −log_10_ adjusted *q* value. Genes with *q* < 0.05 and |log_2_ fold change| > 1 are shown in purple with the core (dark purple) and accessory (light purple) genes indicated. The number of DEGs is given below each image with core only number in parentheses. Histidine metabolism genes are highlighted in teal, and genes involved in alternative sugar metabolism and bacteriocin production are highlighted in red.

**Figure 5.**
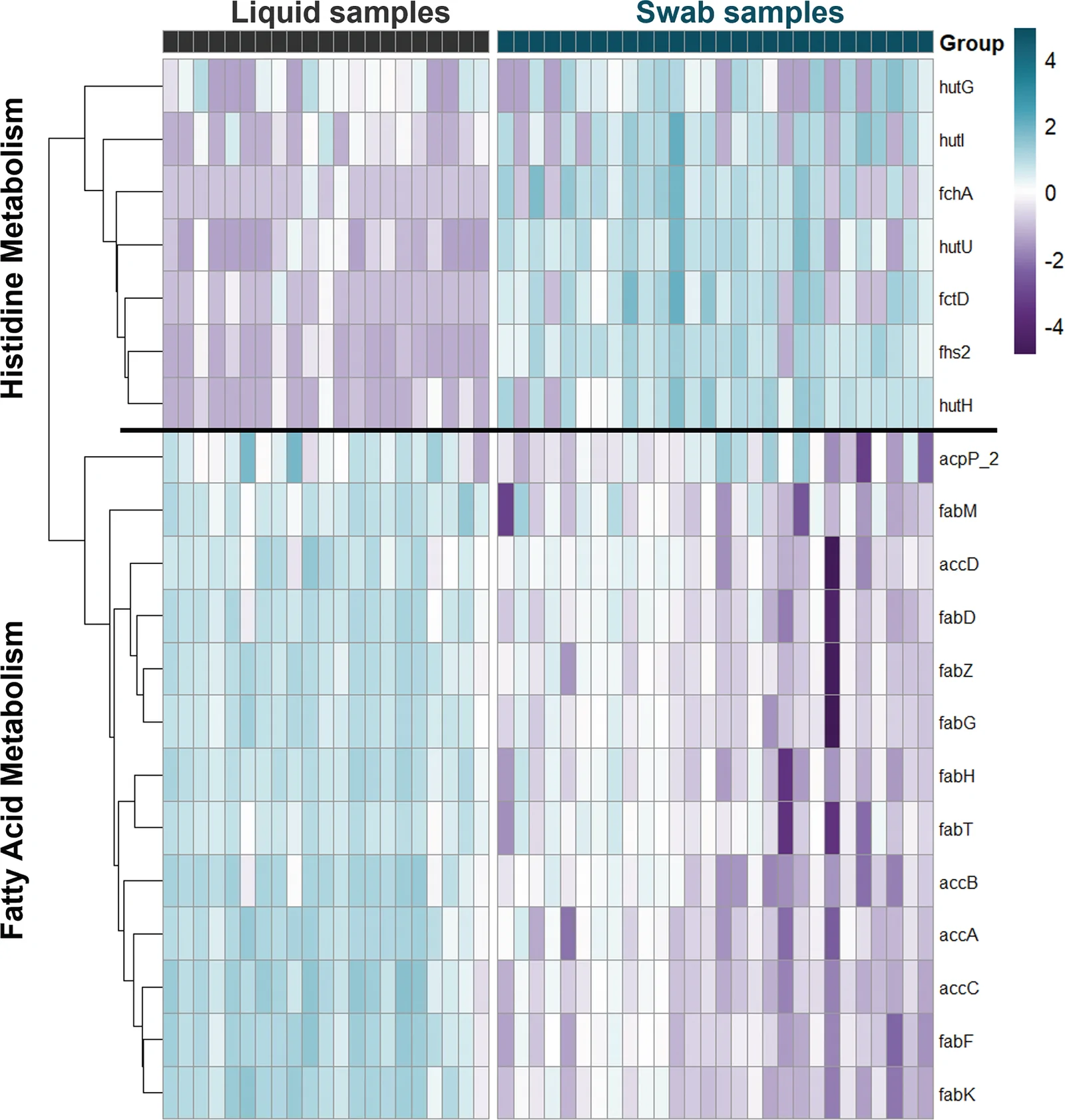
Heatmap of gene expression profiles in histidine and fatty acid metabolism genes Row-scaled expression values for genes involved in histidine metabolism (top) and fatty acid metabolism (bottom). Samples are divided into two groups, liquid (gray, *n* = 21) and swab (visit 1, dark teal, *n* = 28). Gene expression values were standardized as *Z* scores across each gene (row), such that values indicate the number of standard deviations from the gene’s mean expression. Genes within each metabolic group are clustered hierarchically, revealing expression patterns specific to sample types. The two metabolic pathways exhibit distinct expression signatures, highlighting differences in gene regulation between sample groups. The color gradient indicates relative expression levels from low (purple) to high (blue). Data were processed and visualized using R with the heatmap package.

**KEY RESOURCES TABLE:** 

REAGENT or RESOURCE	SOURCE	IDENTIFIER

Bacterial and virus strains		

Group A Streptococcus clinical isolates	This study	Table 1
Group A Streptococcus NZ131	Lab strain/Reference	N/A

Biological samples		

Pediatric pharyngeal swab samples (acute pharyngitis and carrier follow-ups)	Four pediatric clinics in Madison, WI, USA	Protocol #2015-1456-CP016N/A

Chemicals, peptides, and recombinant proteins		

Crystal violet (0.1% solution)	Fisher Scientific	Cat #B2193222
Hyaluronic acid (HA) standards	Fisher Scientific	Cat# 50-186-9545
Stains-all reagent	Fisher Scientific	Cat# AAJ6263003
Trypsin-EDTA	Fisher Scientific	Cat # SM2003C
Triton X-100	VWR	Cat # 97062-208
Lysozyme	VWR	Cat # 97062-138
Mutanolysin	Sigma Aldrich	Cat # SAE0092
TRIzol Max Bacterial Enhancement Reagent	Thermo Fisher	Cat# 16122012
DNA/RNA Shield	Zymo/Fisher Scientific	Cat # 50-444-614

Critical commercial assays		

MIC Test Strips (MTS) for Clindamycin (0.016-256 μg/mL)	Liofilchem	Cat# 92072
MIC Test Strips (MTS) for Amoxicillin (0.016-256 μg/mL)	Liofilchem	Cat# 92021
MIC Test Strips (MTS) for Penicillin G (0.002-32 μg/mL)	Liofilchem	Cat# 92103
MIC Test Strips (MTS) for Erythromycin (0.016-256 μg/mL)	Liofilchem	Cat# 92051
Illumina Nextera Flex DNA Library Prep Kit	Illumina	Cat# 20018704
Nextera DNA CD Indexes	ThermoFisher	Cat# 20018707
Monarch HMW DNA Extraction Kit for Tissue and Bacteria	NEB	Cat# T3060
AMPure PB Beads	PacBio	Cat# 100-265-900
PacBio Elution Buffer	PacBio	Cat# 101-633-500
SMRTbell Express Template Prep Kit 2.0	PacBio	Cat# 100-938-900
SMRTbell Barcoded Adapter Plate 3.0	PacBio	Cat# 102-099-200
TRIzol Max Bacterial RNA Isolation Kit	ThermoFisher	Cat# 16096-040
TURBO DNA-free Kit	ThermoFisher	Cat# AM1907
MICROEnrich Kit	ThermoFisher	Cat# AM1901
MICROBExpress Bacterial mRNA Enrichment Kit	ThermoFisher	Cat# AM1905
Kapa Stranded RNASeq Library Preparation Kit	Roche/Kapa Biosystems	Cat# KK8400
NEBNext Ultra II Directional RNA Library Prep Kit	NEB	Cat# E7765

Deposited data		

Sequenced GAS genomes and RNA-seq datasets	This study	Table 1; NCBI SRA/GEO

Experimental models: Cell lines		

Detroit 562 (Human pharyngeal carcinoma epithelial cells)	ATCC	Cat# CCL-138; RID:CVCL_1171

Experimental models: Organisms/strains		

Human pediatric cohort (ages 5-15 years, acute pharyngitis and pharyngeal carriage)	Four pediatric clinics (Madison, WI, USA)	IRB Protocol #2015-1456-CP016

Oligonucleotides		

TATTGGCTTAGAAAATTAA	Literature cited38,63	*emm* Sense
GCAAGTTCTTCAGCTTGTTT	Literature cited38,63	*emm* Antisense

Software and algorithms		

Porechop v0.2.3	RRID:SCR_016967	Porechop v0.2.3
Flye v2.9	RRID:SCR_017016	Flye v2.9
BWA MEM v0.7.17	RRID:SCR_010910	BWA MEM v0.7.17
Prokka	RRID:SCR_014732	Prokka
Anvi’o v7.x	RRID:SCR_016024	Anvi’o v7.x
pyANI	RRID:SCR_022421	pyANI
trimAl	RRID:SCR_017297	http://trimal.cbgp.upm.es
FastTree	RRID:SCR_015501	http://www.microbesonline.org/fasttree/
MUSCLE v5	RRID:SCR_011812	https://www.drive5.com/muscle/
ape (R package)	RRID:SCR_016201	https://cran.r-project.org/web/packages/ape/
phangorn (R package)	RRID:SCR_017302	https://cran.r-project.org/web/packages/phangorn/
IQ-TREE 2	RRID:SCR_017254	http://www.iqtree.org
Picard MarkDuplicates (v2.x)	RRID:SCR_006525	https://broadinstitute.github.io/picard/
FeatureCounts v2.0.3	RRID:SCR_012919	http://subread.sourceforge.net/
edgeR	RRID:SCR_012802	https://bioconductor.org/packages/edgeR/
ggplot2 (R package)	RRID:SCR_014601	https://ggplot2.tidyverse.org
DIAMOND	RRID:SCR_016071	https://github.com/bbuchfink/diamond

Other		

Covaris g-TUBES	Covaris	Cat# 520079
MagMAX Prime Bead Beating Tubes	ThermoFisher	Cat# A58155
OMNI Bead Ruptor 12	Omni International	Cat# 19-050A

**Table 1.** *Streptococcus pyogenes* isolates with associated sequencing data

Sample name	*emm*	Size (bp)	Coding genes	Genome accession	RNA-seq accession

**Non-carrier (NC) patients (49 total) with isolates and swabs from visit 1 only** Acute infection resolved with antibiotics					
NC-1	4	1,841,636	1,813	SAMN53082547	
NC-2	89	1,730,985	1,670	SAMN53082548	
NC-3	2				
NC-4	4	1,893,450	1,871	SAMN53082549	
NC-5	4	1,897,528	1,908	SAMN53082550	
NC-6	12	1,836,420	1,815	SAMN53082551	
NC-7	28				
NC-8	12	1,754,445	1,700	SAMN53082552	
NC-9	89	1,747,785	1,691	SAMN53082553	
NC-10	75				liquid: GSM9282646 swab1: GSM9282651
NC-11	6	1,788,686	1,774	SAMN53082554	liquid: GSM9282640
NC-12	1	1,864,208	1,837	SAMN53082555	
NC-13	2				
NC-14	1	1,852,244	1,856	SAMN53082556	
NC-15	1	1,853,040	1,858	SAMN53082557	
NC-16	L2.2				
NC-17	12	1,862,991	1,811	SAMN53082558	
NC-18	3				
NC-19	1				
NC-20	2				liquid: GSM9282645 swab1: GSM9282670
NC-21	4	1,896,922	1,908	SAMN53082559	swab1: GSM9282657
NC-22	28				liquid: GSM9282641 swab1: GSM9282665
NC-23	28				
NC-24	3				liquid: GSM9282654 swab1: GSM9282679
NC-25	89	1,721,698	1,583	SAMN53082560	
NC-26	89	1,833,439	1,735	SAMN53082561	
NC-27	89	1,715,094	1,577	SAMN53082562	
NC-28	2				liquid: GSM9282650 swab1: GSM9282677
NC-29	28				liquid: GSM9282653 swab1: GSM9282667
NC-30	3				swab1: GSM9282668
NC-31	12				swab1: GSM9282689
NC-32	1				liquid: GSM9282673 swab1: GSM9282669
NC-33					swab1: GSM9282676
NC-34	2				liquid: GSM9282655 swab1: GSM9282690
NC-35	2				liquid: GSM9282633 swab1: GSM9282642
NC-36	2				swab1: GSM9282652
NC-37	1	1,836,729	1,762	SAMN53082563	liquid: GSM9282680 swab1: GSM9282681
NC-38	28				liquid: GSM9282635 swab1: GSM9282648
NC-39	4				liquid: GSM9282639 swab1: GSM9282656
NC-40	4				liquid: GSM9282649 swab1: GSM9282664
NC-41	4				liquid: GSM9282647 swab1: GSM9282658
NC-42	12				liquid: GSM9282660 swab1: GSM9282661
NC-43	28	1,851,429	1,750	SAMN53082564	swab1: GSM9282675
NC-44	1				liquid: GSM9282636 swab1: GSM9282634
NC-45	4				liquid: GSM9282682 swab1: GSM9282637
NC-46	1				swab1: GSM9282659
NC-47	1				swab1: GSM9282674
NC-48	12				liquid: GSM9282643 swab1: GSM9282666
NC-49	28				liquid: GSM9282644 swab1: GSM9282663
**Carrier (C) patients (13 total) with indicated visit number (–-1 or –-2)** GAS remained following antibiotic treatment and resolution of symptoms					
C1–1	6	1,873,697	1,901	SAMN53082565	
C2–1	4	1,854,376	1,850	SAMN53082566	swab1: GSM9282662
C3–1	89	1,747,469	1,698	SAMN53082567	
C3–2	89	1,747,469	1,698	SAMN53082568	swab2: GSM9282687
C4–1	6	1,926,547	1,987	SAMN53082569	swab1: GSM9282683
C4–2	6				swab2: GSM9282686
C5–1	89	1,729,940	1,669	SAMN53082570	swab1: GSM9282684
C5–2	89				swab2: GSM9282685
C6–1	1	1,835,818	1,759	SAMN53082571	
C7–1	89	1,745,579	1,691	SAMN53082572	
C8–1	89	1,779,818	1,728	SAMN53082573	
C9–1	4	1,887,610	1,824	SAMN53082574	
C10–1	6	1,933,860	1,997	SAMN53082575	
C11–1	28				
C12–1	6				liquid: GSM9282638 swab1: GSM9282678
C13–1	6	1,839,146	1,795	SAMN53082576	swab1: GSM9282672
C13–2	6	1,839,406	1,795	SAMN53082577	swab2: GSM9282671

Genomes used for comparison					

SF370	1	1,852,433	1,710	NC_002737.2	
MGAS10750	4	1,937,111	1,755	NC_008024.1	
MGAS10394	6	1,899,877	1,754	NC_006086.1	
MGAS9429	12	1,836,467	1,668	NC_008021.1	
H293	89	1,726,248	1,692	HG316453.2	

**Table 2.** Carrier-only amino acid polymorphisms

Isolate	Protein	Close loci	AAP(s)

C1	ScpA	M6_Spy1718	L1144S
	SpeB	M6_Spy1735	Q181 deletion^*a^
	Slo	M6_Spy0188	F301 deletion
C2	FruA	MGAS10750_Spy0753	Q54R
	CynT	MGAS10750_Spy0196	Q12Y
	Pip	MGAS10750_Spy1947	S243L
C3	PacL	Spy_0623	A383V
	FbpA	WP_031577373	P193_S194insETP
	CitF	Spy_1189	A350T
C4	ScpA	M6_Spy1718	D420N
	DltD	M6_Spy1040	S364F
C5	DltA	M6_Spy1043	Q193H
	CovS	Spy_0337	K123N
C6	PepF	Spy_0606	G356D
	HasA	Spy_2200	Nt deletion resulting in frameshift after first 7 amino acids. May start at M25
C7	Ska	Spy_1979	N196G, S202L, K204R, E205N
	LeuS	Spy_0173	F3L
	AroF	Spy_0810	K83E
	Ccs4	Spy_2111	Y218C
C8		M6_Spy0849	deletion I131
C9	HsdR	MGAS10750_Spy1676	V309I, L612F
	Sof	MGAS10750_Spy1823	Nt deletion resulting in frameshift after E334.
	LctO	MGAS10750_Spy0338	deletion after G198 leading to truncation
	SpyCEP	MGAS10750_Spy0339	E635K
	hypothetical	MGAS10750_Spy0391	A31T
	DltB	MGAS10750_Spy1165	deletion after I31 leading to trunction
C10	DltD	M6_Spy1043	S364F
C13	ClfA	M6_Spy0159	Nt insertion causing premature trunction after L223
	hypothetical	M6_Spy0377	Nt deletion resulting in frameshift after first 6 amino acids. May start at M25
	RecB	SpyM6JRS4_02920	E242K
	hypothetical	M6_Spy0764	V237G
	hypothetical	M6_Spy1172	Nt deletion resulting in frameshift after E64
	UgpE	M6_Spy1327	K3Q
	SclA	M6_Spy1695	NT insertion causing frameshift after F79

a* Normal SpeB activity per casein milk agar plate assay.

## Highlights

- Direct *in vivo* RNA profiling of GAS from human oropharyngeal swabs
- Acute infection and post-treatment carriage display distinct expression profiles
- Carrier and non-carrier strains share similar genotypes and *in vitro* phenotypes
- Acute-phase bacterial transcriptional profiles do not predict treatment failure
