# Supplementary material for: Streptococcus pyogenes carriage is associated with distinct in vivo transcriptional states but not carrier-specific genotypes
Source: Cell Rep. Author manuscript; Available in PMC 2026 Oct 15. (PMC13671490; doi:10.1016/j.celrep.2026.117925)
Supplement: Supplementary file 1 [file NIHMS2212251-supplement-1.pdf]

Cell Reports, Volume *45*

Supplemental information

Streptococcus pyogenes carriage is associated with distinct *in vivo* transcriptional states but not carrier-specific genotypes

Nicholas Faiola, Jason M. Wood, Nicole Casab, May Xu, Kevin Quirk, Christopher Thomas, Eliza Klos, Jordan Thesier, Mitchell Waldran, Mark Maienschein-Cline, Michael J. Federle, Gregory P. DeMuri, Ellen R. Wald, and Laura C. Cook

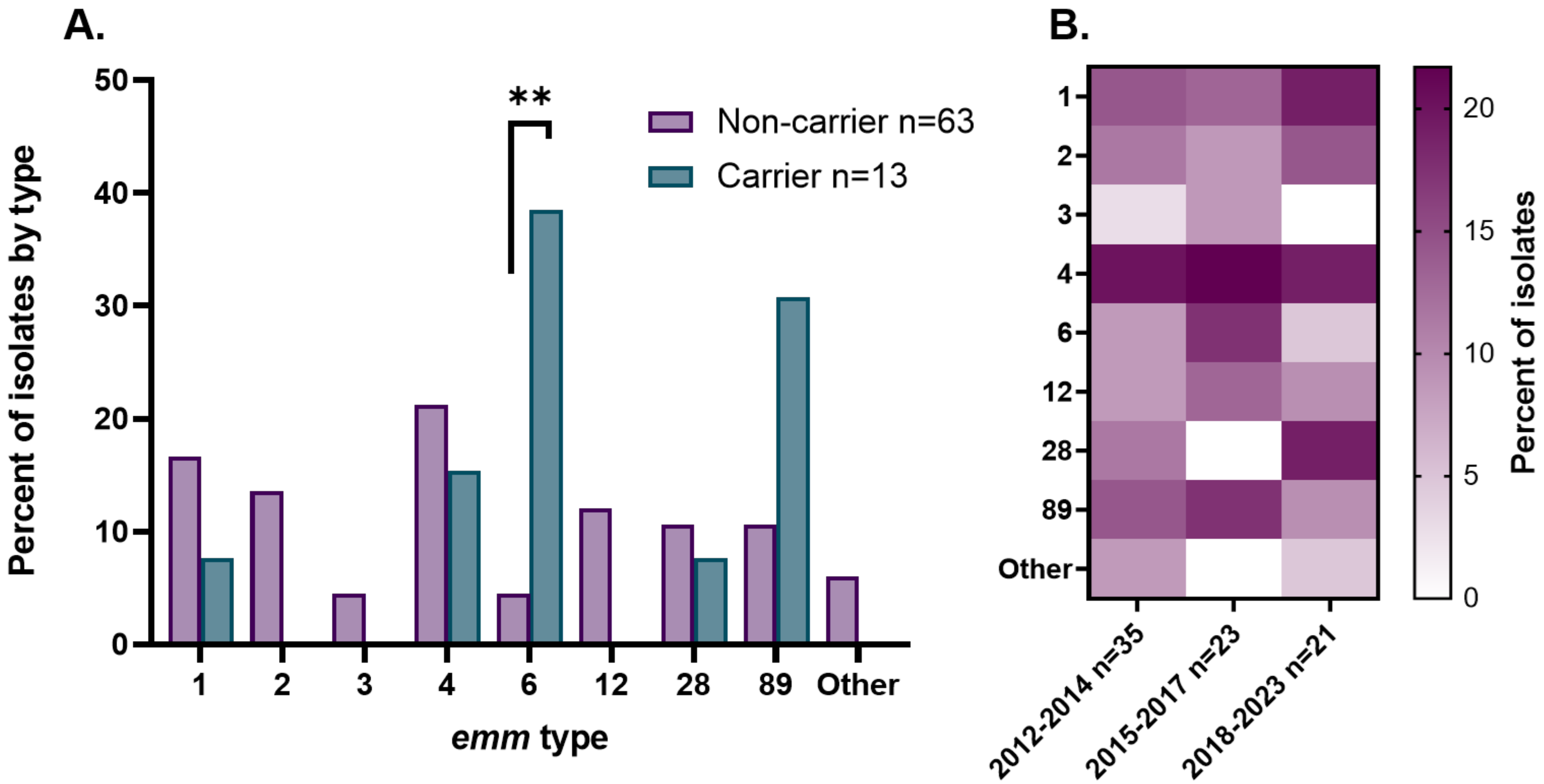

Figure S1. *emm* type of acute and carrier GAS strains.
(A) *emm* type distribution in non-carrier (purple) and carrier (blue) isolates. (B) Distribution of *emm* types grouped by year of collection. Darker shade represents a higher percentage of a particular *emm* type in those year blocks. Fisher's exact test was used to determine statistical significance. ** p value < 0.01.

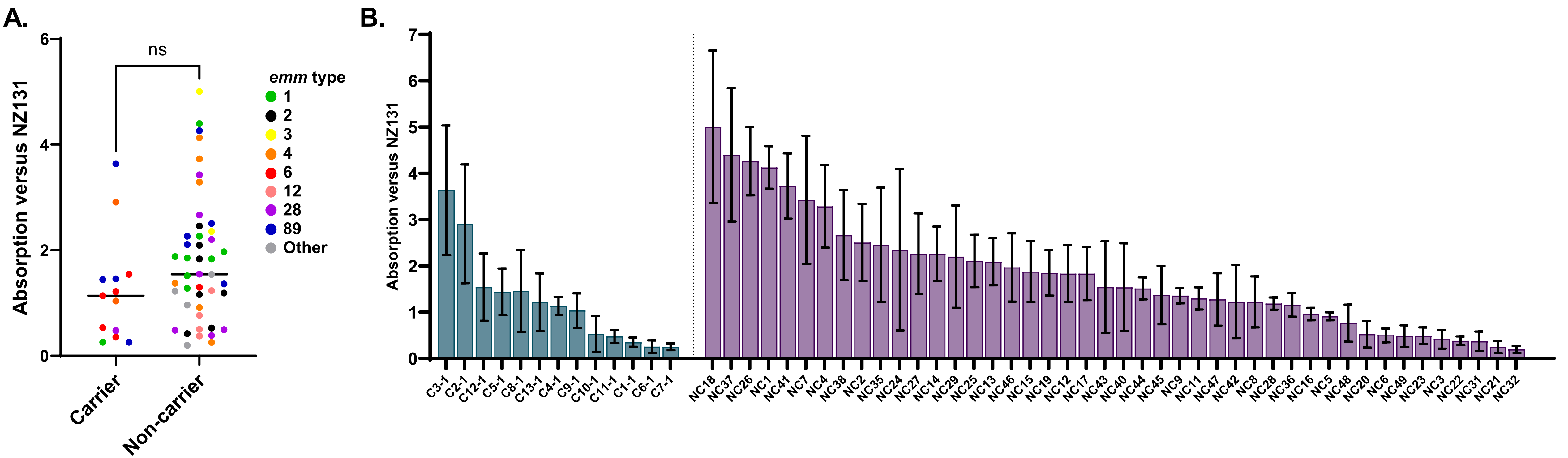

Figure S2. *in vitro* biofilm formation is not associated with carriage propensity.
Biofilm formation on abiotic surfaces was measured using crystal violet staining. Crystal violet absorption is given as a ratio to GAS strain NZ131 which was used as an internal control for each assay. The absorbance by group is shown (**A**) with different *emm* types labeled in different colors. Value s for all individual isolates relative to an internal NZ131 control are also shown (**B**). At least three biological replicates and two technical replicates were used for each data point. The average and standard deviation are shown.

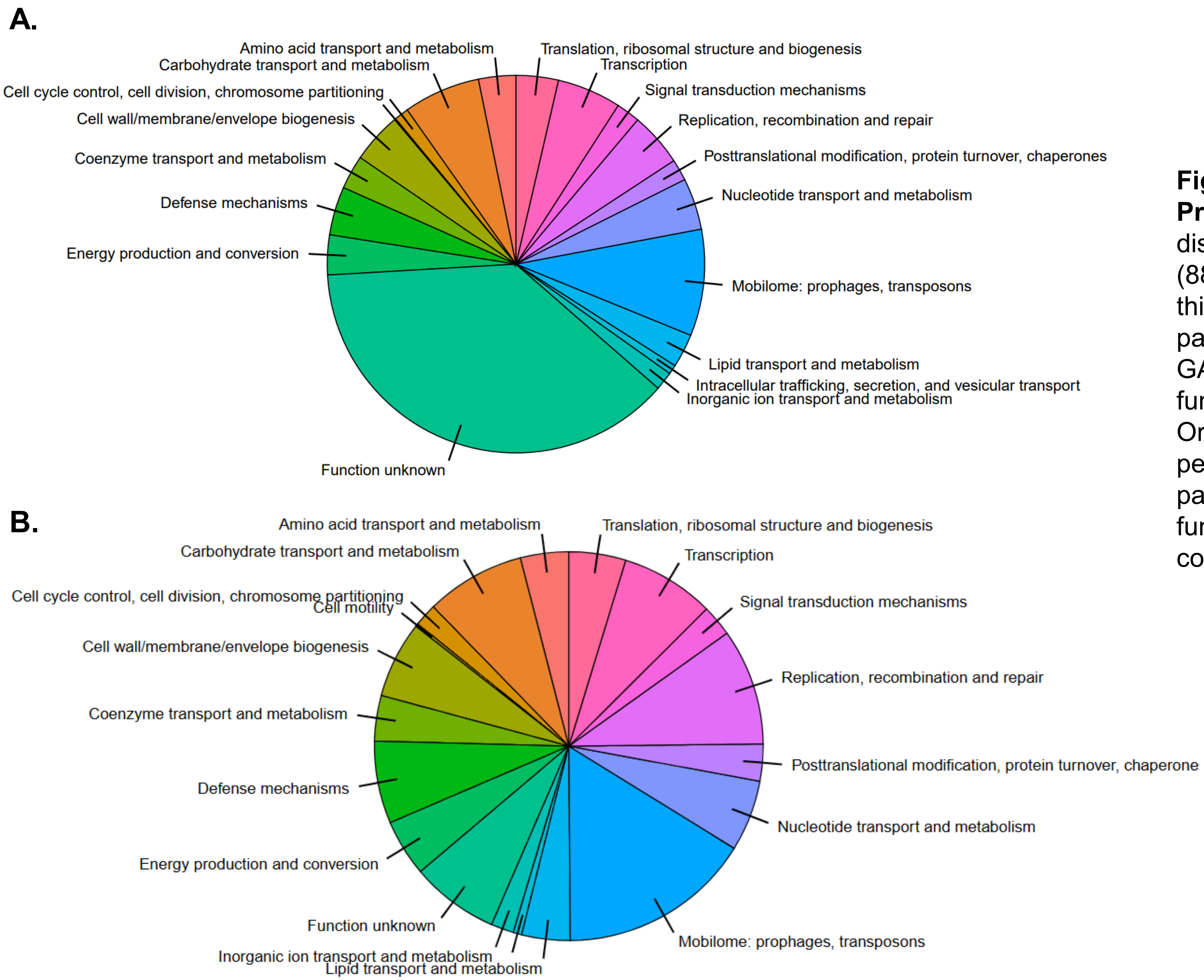

Figure S3. Functional Classification of Predicted Genes via COG Analysis. The distribution of genes in the accessory genome (889 genes) of the GAS isolates sequenced in this study (n=31) (A) and of the transcriptomic pan-genome (1,169 genes) made up of eight GAS reference genomes (B) into various functional categories based on the Clusters of Orthologous Groups (COG) database. A high percentage of the accessory content for both pan-genomes is made up of genes with unknown function or those found in the mobilome containing phages and transposons.

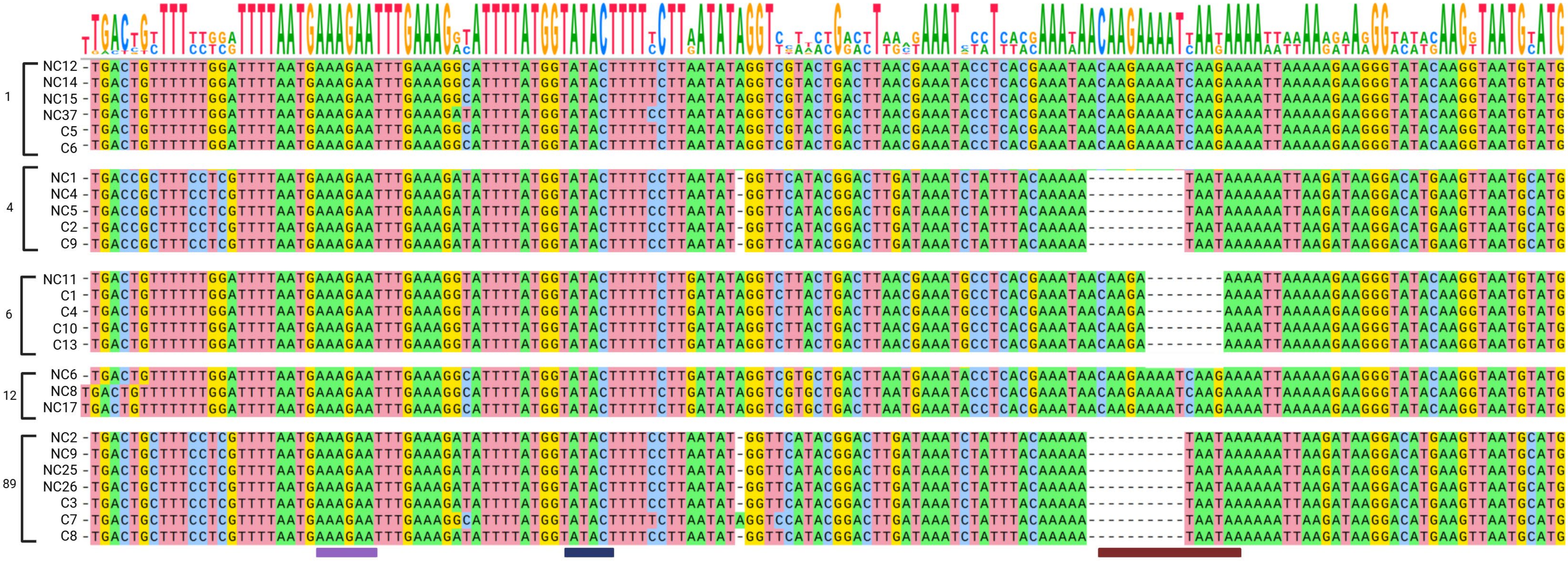

Figure S4. Alignment of the *mga* promoter region across sequenced isolates.
Multiple sequence alignment of the *mga* promoter region from sequenced isolates. Purple and dark blue boxes denote the predicted −35 and −10 promoter elements, respectively. The maroon bar indicates regions of deletion that are conserved by *emm* type among these isolates.

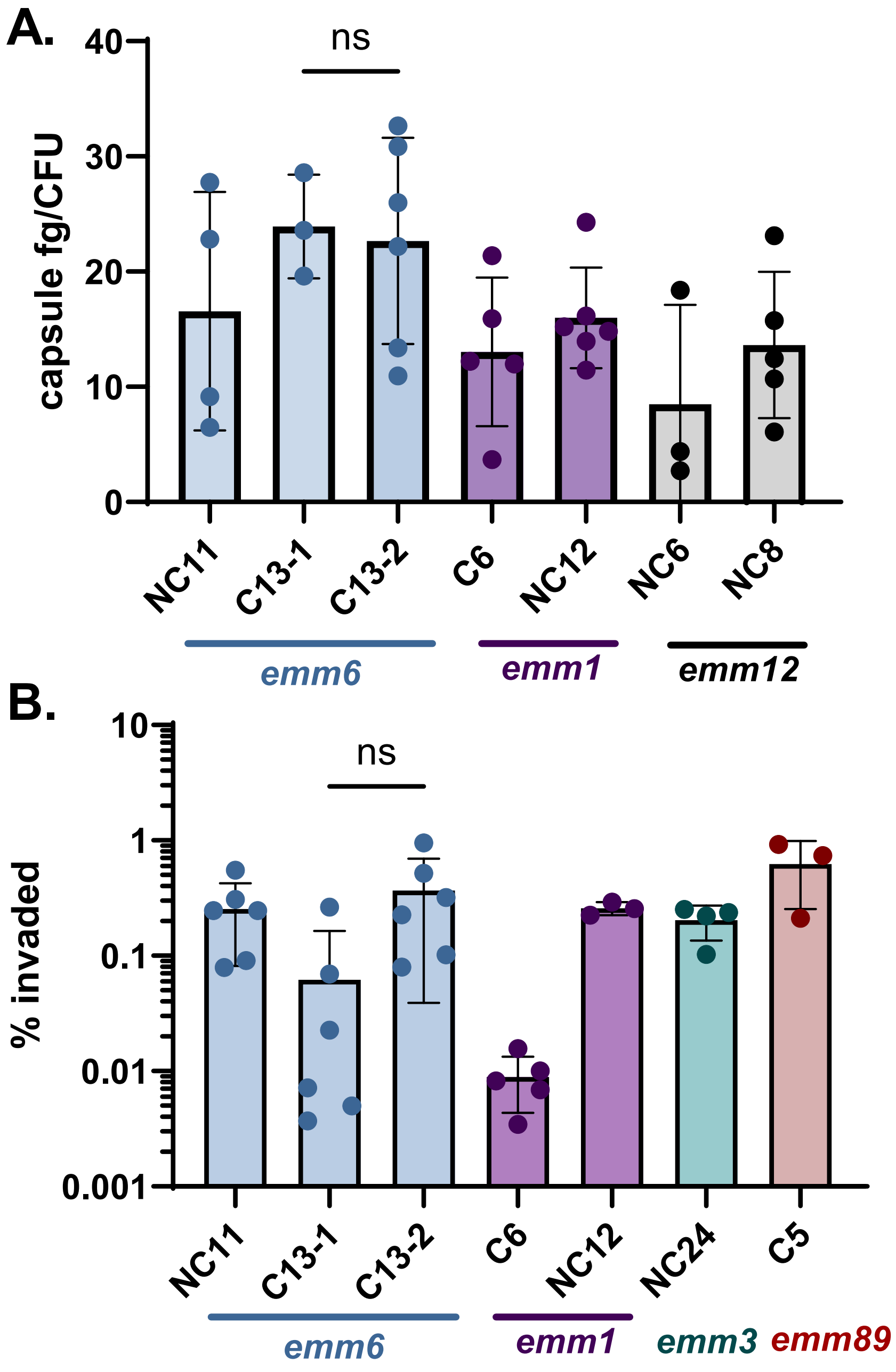

Figure S5. Capsule production and invasion in selected isolates.
(**A**) Quantification of capsule production expressed as femtogram (fg) per CFU. (**B**) Intracellular invasion efficiency into Detroit 562 epithelial cells expressed as a percentage of initial inoculum. GAS isolates are grouped by *emm* type. Biological replicates (n=3-6) are shown and bars represent the mean and standard deviation. ns = not significant using paired *t* test to compare C13 isolates from visits 1 and 2.

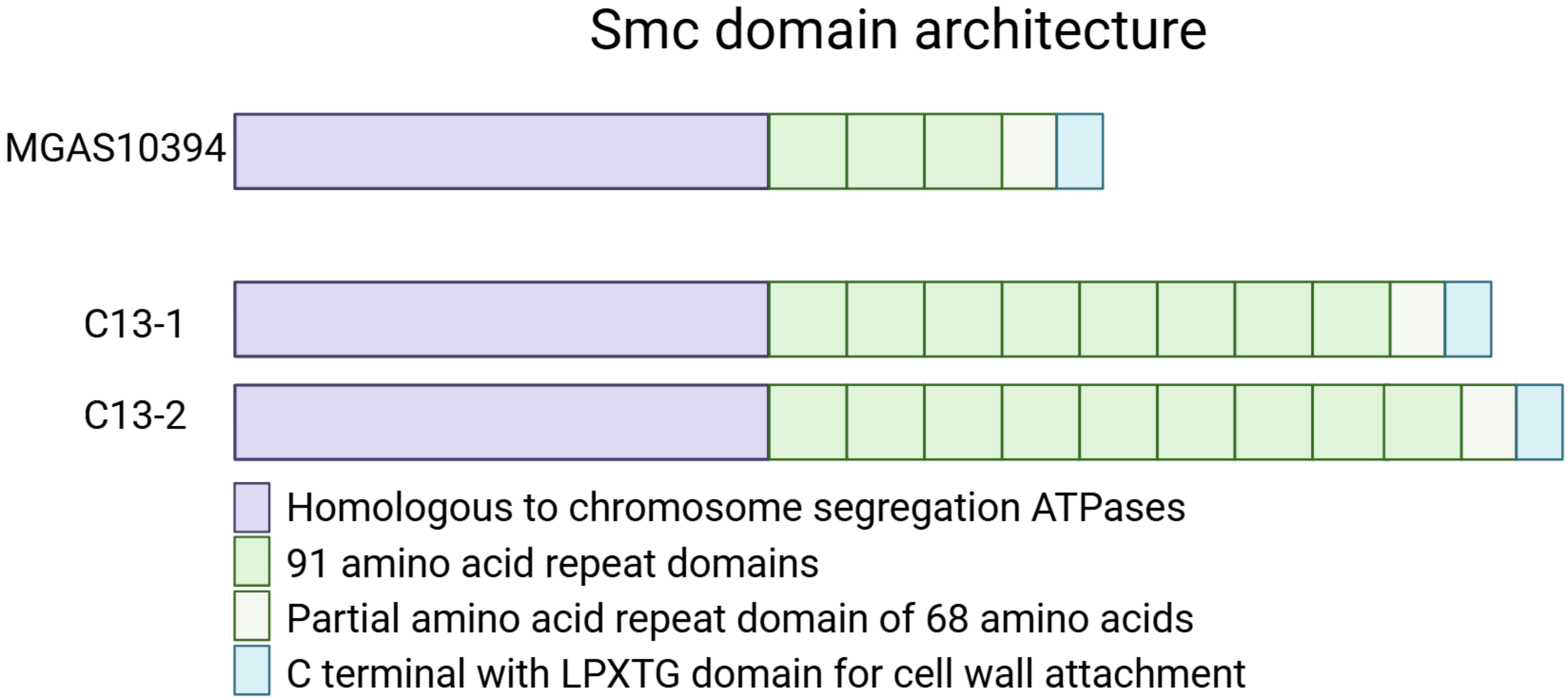

Figure S6. Domain architecture of Smc. The closest homolog to Smc is M6_Spy1173 from MGAS10394. Smc is found in C13-1 and C13-2 but the number of repeat domains differs between the three strains as shown. The purple region of the protein is predicted homologous to a chromosome segregation ATPase while the green and blue sections are predicted to be surface exposed adhesins with an associated LPXTG domain in the blue region.

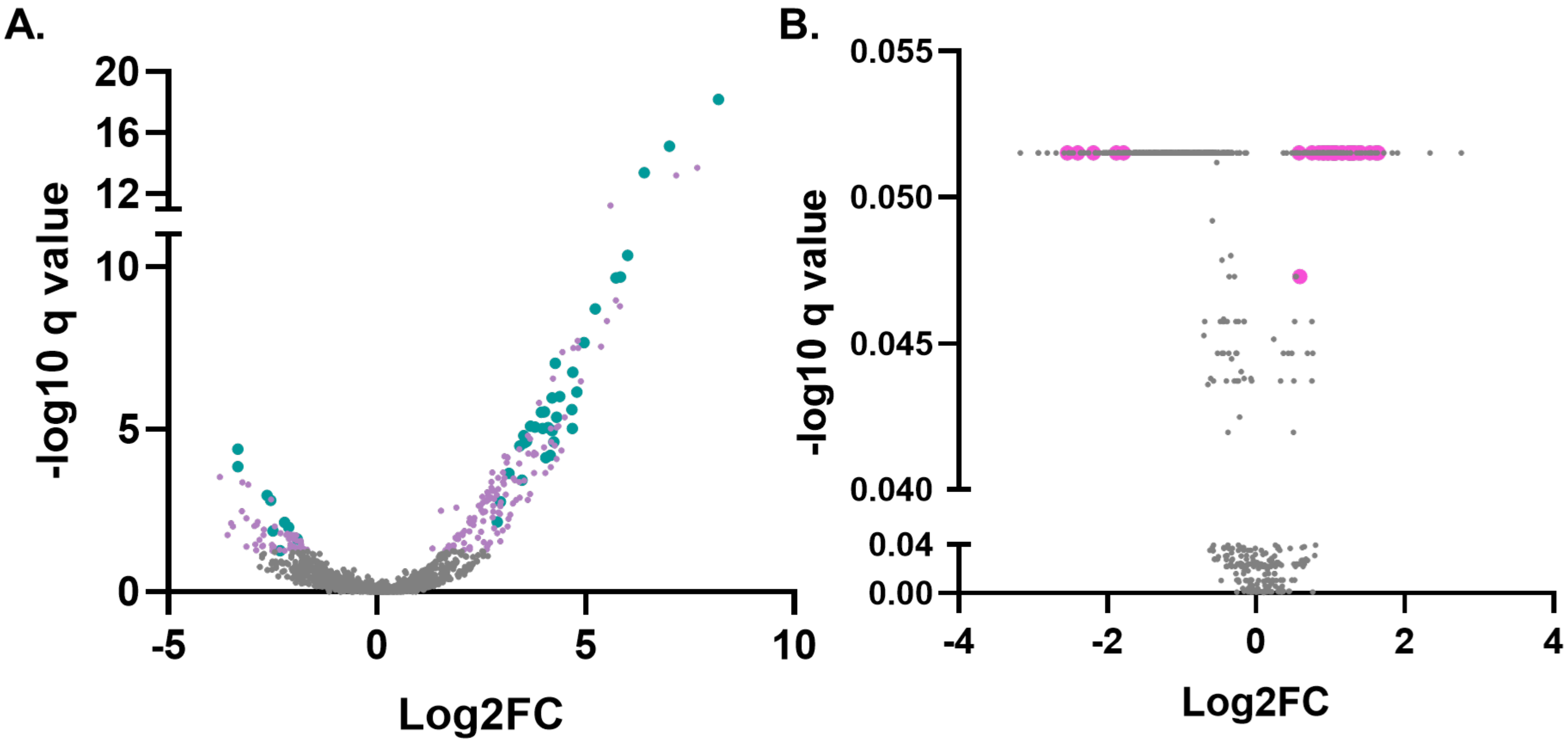

Figure S7. Differential gene expression in matched swab sets.
(A) Volcano plot comparing four *emm28* isolates (NC22, NC29, NC38 and NC49) grown in liquid culture with their matched swab samples. Genes are plotted by $\log_2$ fold change (Log2FC) and $-\log_{10}$ q value. Gray points denote genes that did not reach statistical significance, purple points denote statistically significant genes, and teal points indicate the top 50 most significant genes identified in the bulk grouped dataset (Fig. 4a and Table S4). **(B)** Volcano plot comparing three longitudinal swab sets at visit 1 versus visit 2. Grey points denote genes that did not reach statistical significance, pink points indicate the top 33 most significant genes identified in the bulk grouped dataset (Fig. 4b and Table S6).

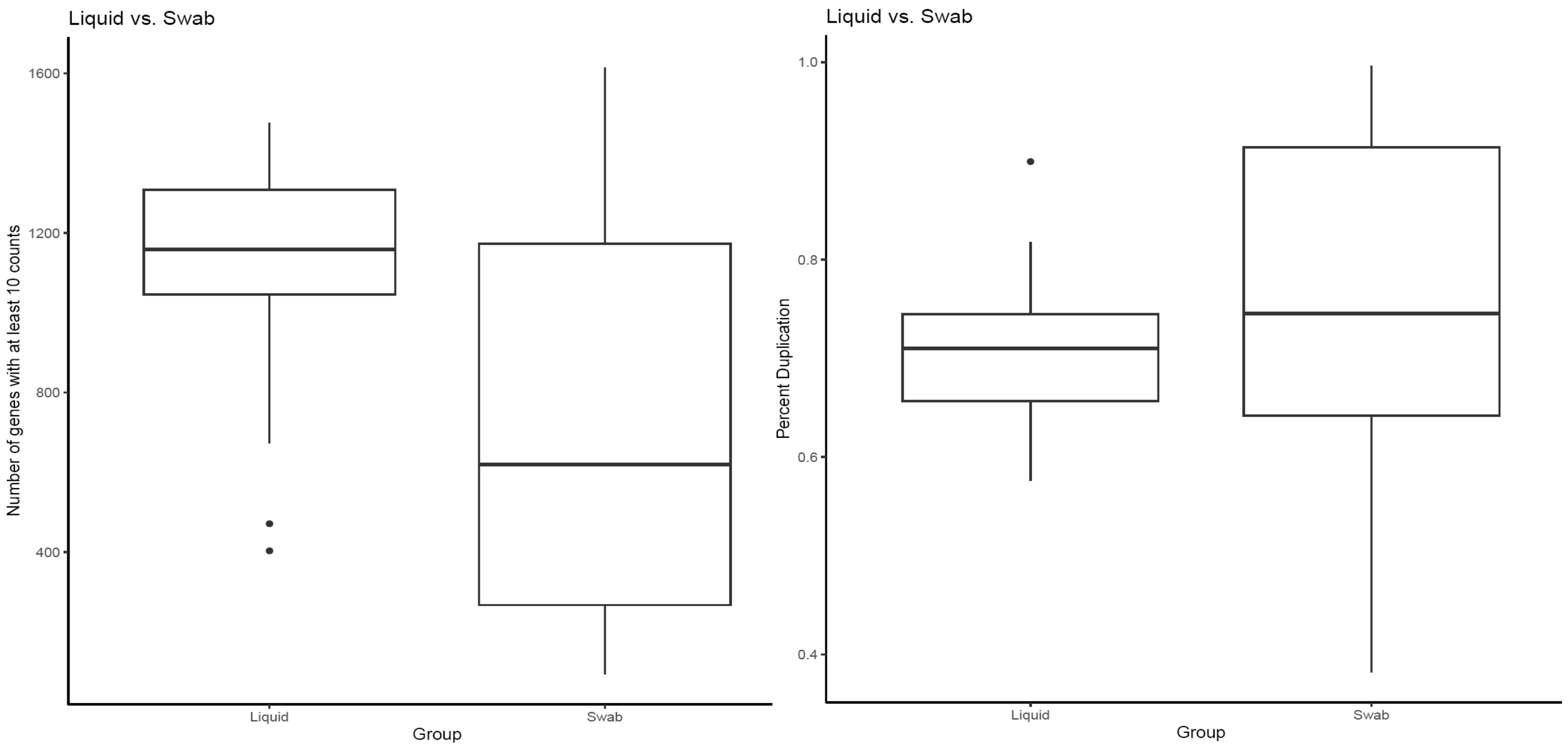

Figure S8. QC statistics from RNA-seq data. Boxplots show the number of genes with at least 10 counts (left) and percent duplication for liquid and swab samples (right). In each boxplot, the center line represents the median, boxes indicate the interquartile range (IQR), whiskers extend to 1.5× the IQR, and points denote outliers. As expected, liquid samples generally exhibit higher gene detection and lower duplication rates compared to *in vivo* swab samples.
